# One-Drop Serum Screening Test to Monitor Tissue Iron Accumulation

**DOI:** 10.1021/acs.analchem.5c00778

**Published:** 2025-05-30

**Authors:** Gabriely S. Folli, Anne Louise S. Torres, Matthews Martins, Luiz Ricardo Rodrigues Silva, Vinícius Bermond Marques, Maria Tereza Carneiro, Larissa Dias Roriz, Leonardo dos Santos, Wanderson Romão, Francis L. Martin, Paulo R. Filgueiras, Valério G. Barauna

**Affiliations:** † Departamento of Chemistry, 28126 Federal University of Espírito Santo (UFES), Av. Fernando Ferrari, 514, Vitória, Espírito Santo 29075-910, Brazil; ‡ Department of Physiological Sciences, Federal University of Espírito Santo (UFES), Av. Marechal Campos, 1468, Maruípe, Vitória, Espírito Santo 29040-090, Brazil; § Federal Institute of Education, Science, and Technology of Espírito Santo, Av. Ministro Salgado Filho, 1000, Vila Velha, Espírito Santo 29106-010, Brazil; ∥ Department of Cellular Pathology, Blackpool Teaching Hospitals NHS Foundation Trust, Whinney Heys Road, Blackpool FY3 8NR, U.K.

## Abstract

Although iron is an essential element for vital body functions, iron overload (IO) is accompanied by significant cellular damage due to its accumulation within organs. Thus, early diagnosis and accurate identification of the affected organs are critical for preventing irreversible damage and improving patient survival rates. Diagnosing tissue iron deposits relieves invasive biopsies with atomic absorption spectrometry (reserved for specific cases) or noninvasive but costly and time-consuming imaging techniques like computerized tomography and magnetic resonance, which provide limited analytical data and are unsuitable for routine screening. As an alternative, Fourier transform infrared spectroscopy combined with machine learning has emerged as a promising approach for supporting medical decision-making. In this study, we developed a minimally invasive method to identify IO and quantify iron levels in blood and tissues (heart, liver, spleen, and kidney) without biopsies. PLS-DA classification models and PLS regression models were constructed based on samples categorized into a control group (*n* = 10) and three iron-administered groups at 250 mg kg^–1^ (*n* = 14), 500 mg·kg^–1^ (*n* = 13), and 1000 mg·kg^–1^ (*n* = 15). Iron levels were measured in blood samples and tissue biopsies (spleen, heart, liver, and kidney). The binary classification models (control vs iron-administered) and multiclass models (control, 250, 500, and 1000 mg·kg^–1^) demonstrated satisfactory performance into train and validation groups. PLS regression models for quantifying iron concentrations in blood and tissues exhibited excellent linearity and low associated errors across both calibration and test groups. Permutation tests confirmed that all models found a real class structure in the data, were not random, and were built using true chemical information. The chemical insights from the spectra may reflect adaptations associated with iron-induced dysregulation. Alterations in biomolecules could reflect systemic stress responses and may result from free radicals generated by the iron-induced Fenton reaction. Moreover, key spectral regions revealed functional interrelationships, particularly between spleen and liver, and heart and kidneys. In summary, the findings support the potential of this innovative for future research to identify IO and quantify iron levels in human blood and different human tissues using only a single drop of blood without tissue biopsies.

## Graphical abstract

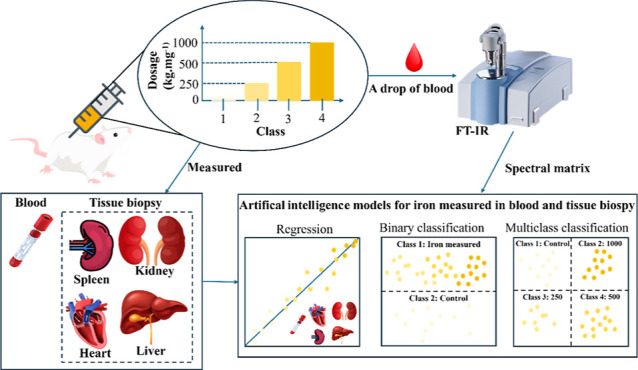

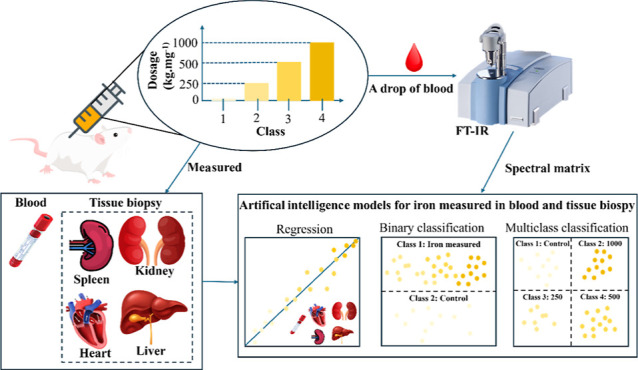

## Introduction

Iron is an essential mineral for maintaining body homeostasis and is involved in metabolic processes such as DNA synthesis, electron transport in the mitochondria, and primarily in oxygen transport through its role in hemoglobin.^1^ However, as there are no well-developed mechanisms to control iron excretion, the excessive administration can overload the organism with significant morbidity and mortality. Excess free iron in the body catalyzes chemical reactions, generating reactive oxygen species (ROS) that damage macromolecules, including proteins, DNA, and lipids, as well as organelles such as lysosomes and mitochondria, ultimately affecting tissues and organs.^2^

Thus, the signs and symptoms of iron overload (IO) depend on the damage resulting from its accumulation in organs such as the liver, heart, spleen, muscles, endocrine glands, and bone marrow, with the liver being the leading site of deposition.^3–6^ Iron-loading conditions primarily manifest as conditions such as heart failure, arrhythmias, and cirrhosis.^7^ For this reason, determining the iron accumulation in vital organs is crucial to guiding patient care. Therefore, early diagnosis and awareness of the affected organs are essential for preventing irreversible damage and improving the survival of patients with IO.

Currently, tissue deposits associated with systemic IO are determined using atomic absorption spectrometry on tissue biopsy samples.^5^ This method has several drawbacks, as it is invasive, expensive, and time-consuming, and results depend heavily on the analyst’s qualifications, making it surgery- and laboratory-dependent. Consequently, Fourier transform infrared (FTIR) spectroscopy combined with machine learning has emerged as a promising tool for medical decision-making.^8–11^ This technique is lab-independent, cost-effective, and provides rapid results.^12^ FTIR spectroscopy measures the interaction of mid-IR radiation with matter across different wavelengths. It is possible due to molecular vibrations that occur with changes in the dipole moments of the chemical bonds involved in the interaction, allowing for chemical information to be obtained at the molecular level of biomolecules present in biofluids such as saliva, plasma, serum, and urine.^13–16^

In previous studies by our group, Leal et al. (2021)^17^ developed an initial classification model for diagnosing acute IO using plasma samples. By applying both unsupervised and supervised methods, we achieved 100% accuracy (ACC). Additionally, the study identified a list of potential biomolecules associated with detected vibrational modes. In the present study, we developed a one-drop FTIR-based method to identify IO and quantify iron levels in blood and tissue without requiring invasive biopsies. To this aim, we created classification models with multivariate analysis to differentiate between normal and IO samples and regression models to quantify IO in different organs.

## Experimental Section

The study was conducted by using rats with varying levels of iron intoxication. Blood samples and tissue biopsies (spleen, heart, liver, and kidney) were collected to identify and quantify the iron levels. Machine learning models were developed based on FTIR spectra obtained during the analysis. A schematic representation of the complete experimental procedure is provided in Figure 1.

**Figure 1:**
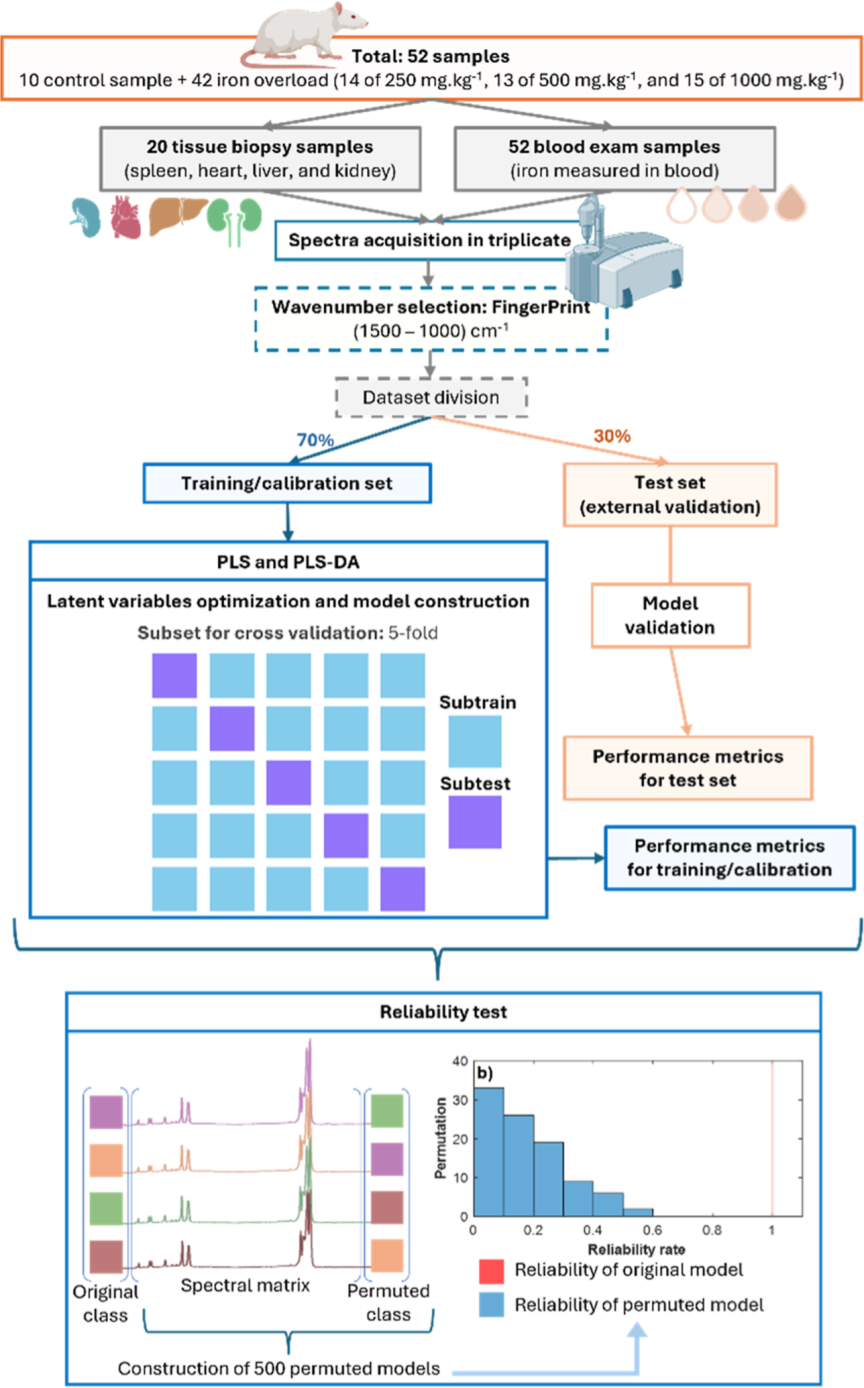
Flowchart for obtaining artificial intelligence models.

### Animal Model of Iron Overload

Male Wistar rats (250–300 g) were obtained from the Health Sciences Centre, Federal University of Espirito Santo animal facility. Animals were housed under controlled temperature (approximately 25 °C) and a 12 h light–dark cycle, with ad libitum access to water and rodent chow. All animal procedures were approved by the Institutional Animal Care and Use Committee (Protocol #51/2019, CEUA-UFES) and adhered to the ethical principles outlined in the Brazilian Guidelines for the Care and Use of Animals for Scientific and Teaching Purposes. Acute IO was induced by a single intraperitoneal injection of iron-dextran (Ferrodex 10%, Fabiani Saúde Animal Ltda, São Paulo, Brazil) as previously described by Lucesoli et al. (1999)^3^ and Rossi et al. (2016).^18^ A total of 52 animals were randomly divided into control (*n* = 10) and three groups injected with iron-dextran: 250 mg·kg^–1^ (*n* = 14), 500 mg·kg^–1^ (*n* = 13), and 1000 mg·kg^–1^ (*n* = 15). All animals received the same total injection volume per body weight by adjusting the saline volume.

### Euthanasia and Sample Collection

24 h after the iron administration, blood was collected by aorta puncture with animals under general anesthesia induced by an intraperitoneal injection of ketamine (100 mg·kg^–1^, 2%) and xylazine (10 mg·kg^–1^, 10%). After euthanasia by exsanguination, the liver, spleen, heart, and kidneys were collected and rapidly frozen at −20 °C. Blood was centrifuged at 4 °C and 1066*g* for 20 min to obtain serum and then stored at −20 °C.

### Serum and Tissue Iron Measurement

Serum iron analysis was conducted on an automatic photometric reader, a Winer CMD600. Tissue iron was determined by inductively coupled plasma optical emission spectrometry (ICP-OES) (Optima 7000DV, PerkinElmer, USA) at the Department of Chemistry, Federal University of Espírito Santo. Samples were dried at 60 °C for 72 h, ground, and digested in a mixture of nitric acid, hydrogen peroxide, and ultrapure water using a microwave digestion system. After digestion, the samples were diluted and analyzed by ICP-OES. Yttrium was used as an internal standard for calibration. The limit of quantification was 2.79 mg·kg^–1^. ACC was assessed by analyzing a certified reference material (MR 05/12: Bovine Liver Tissue, Embrapa).

### Spectral Analysis

The equipment utilized for the mid-IR spectral acquisition was the Alpha II Compact FTIR spectrometer (Bruker Optics, Ettlingen, Germany) operated by OPUS 5.5 software and an attenuated total reflection (ATR) diamond crystal. The spectral range was from 4000 to 400 cm^–1^, acquired in absorbance mode with 4 cm^–1^ resolution, with 32 scans for the background and sample. The diamond-sampling window was cleaned with ultrapure water (Milli-Q) and 70% ethanol v/v for each measurement, and after each triplicate sequence of a sample, the background function was performed. 20 μL of serum was used for each sample in triplicate (*n* = 156 spectra) after at least 2 h of drying and transferred onto the ATR diamond crystal for spectral acquisition.

### Univariate Analysis

The Shapiro–Wilk test was employed to assess the normality of the distribution within each group of iron concentrations (see Table 1 and Figure S1). Mean comparison tests were subsequently performed based on the characteristics of the sample distributions. Specifically, an unpaired student’s *t*-test was applied to distributions that met the normality assumption, while the nonparametric Mann–Whitney test was utilized for distributions that deviated from normality. Statistical significance was considered when *p* < 0.05.

**Table 1:** Iron Measurement Classes in Blood, Spleen, Heart, (d) Liver, and (e) Kidney^a^

class	control	250 mg·kg^–1^	500 mg·kg^–1^	1000 mg·kg^–1^
blood	215 ± 30	2920 ± 468*	6085 ± 1646*	16914 ± 5077*
heart	337 ± 59	759 ± 93*	1346 ± 270*	2316 ± 355*
liver	253 ± 79	5712 ± 744*	7472 ± 544*	9885 ± 710*
spleen	1903 ± 1623	11415 ± 2267*	13977 ± 2008*	18708 ± 2787*
kidney	213 ± 77	664 ± 157*	968 ± 251*	2391 ± 618*

a Data represents the mean ± standard deviation of the mean. *p < 0.05 vs control.

### Machine Learning

The acquired spectra were randomly divided into two subgroups: training/calibration and test.^19^ The division was performed independently for each class to ensure that the same proportion of the training and test sets was maintained with all replicas remaining within the same partition group. Subsequently, classification models (binary PLS-DA and multiclass PLS-DA) and a regression model (PLS) were developed by using the training/calibration samples. The training/calibration samples were used for cross-validation and model construction, and 30% named in the test were used for model validation (see Figure 1). The spectra were processed by different methods (standard normal variate—SNV,^20^ multiplicative scatter correction—MSC,^21^ first derivative, second derivative, airPLS,^22^ and Savitzky–Golay^23^) and their combinations (Tables S1–S5).

Binary PLS-DA classification models were constructed to differentiate between iron-treated groups (250, 500, and 1000 mg·kg^–1^) and the control group. Additionally, two types of multiclass PLS-DA models were developed. The first multiclass model included all four iron levels: class 1 (control), class 2 (250 mg·kg^–1^), class 3 (500 mg·kg^–1^), and class 4 (1000 mg·kg^–1^). The second multiclass PLS-DA model grouped the iron levels into three classes: class 1 (control), class 2 (250 and 500 mg·kg^–1^), and class 3 (1000 mg·kg^–1^). Following spectral acquisition, cross-validation was performed with the *k*-fold approach (*k* = 5) to optimize the number of latent variables (LVs). Following LV optimization, the models were created by using the training set and validated by using the test samples.

Performance metrics were generated from the model predictions for the classification/regression models in both the training/calibration and test sets (Figure S1). The binary and multiclass PLS-DA classification models were assessed through performance parameters for classification models: ACC, sensitivity (sens.), specificity (spec.), false-positive rate (FPR), and false-negative rate (FNR).^24–26^ The PLS regression models were evaluated based on regression model performance parameters for calibration (root-mean-square error of calibration—RMSEC, linearity of calibration—*R*_c_^2^, and limits of detection—LoD, and quantification—LoQ) and test (root-mean-square error of test—RMSEP and linearity of test—*R*_p_^2^) sets. Permutation models were developed to assess the competence of the classifier. For classification models, reliability rates were evaluated (Figures 2, 4 and 5), while regression models were estimated using RMSE (Figure 7). Essentially, the permutation test procedure measures how likely the observed metrics would be obtained by chance.^27^ The permutation test used 500 models generated from the original vector class permutations. This approach aimed to compare the reliability of the permuted models with the original model, evaluating the robustness and reliability of the original models and verifying their statistical significance in identifying or quantifying the target class.

**Figure 2:**
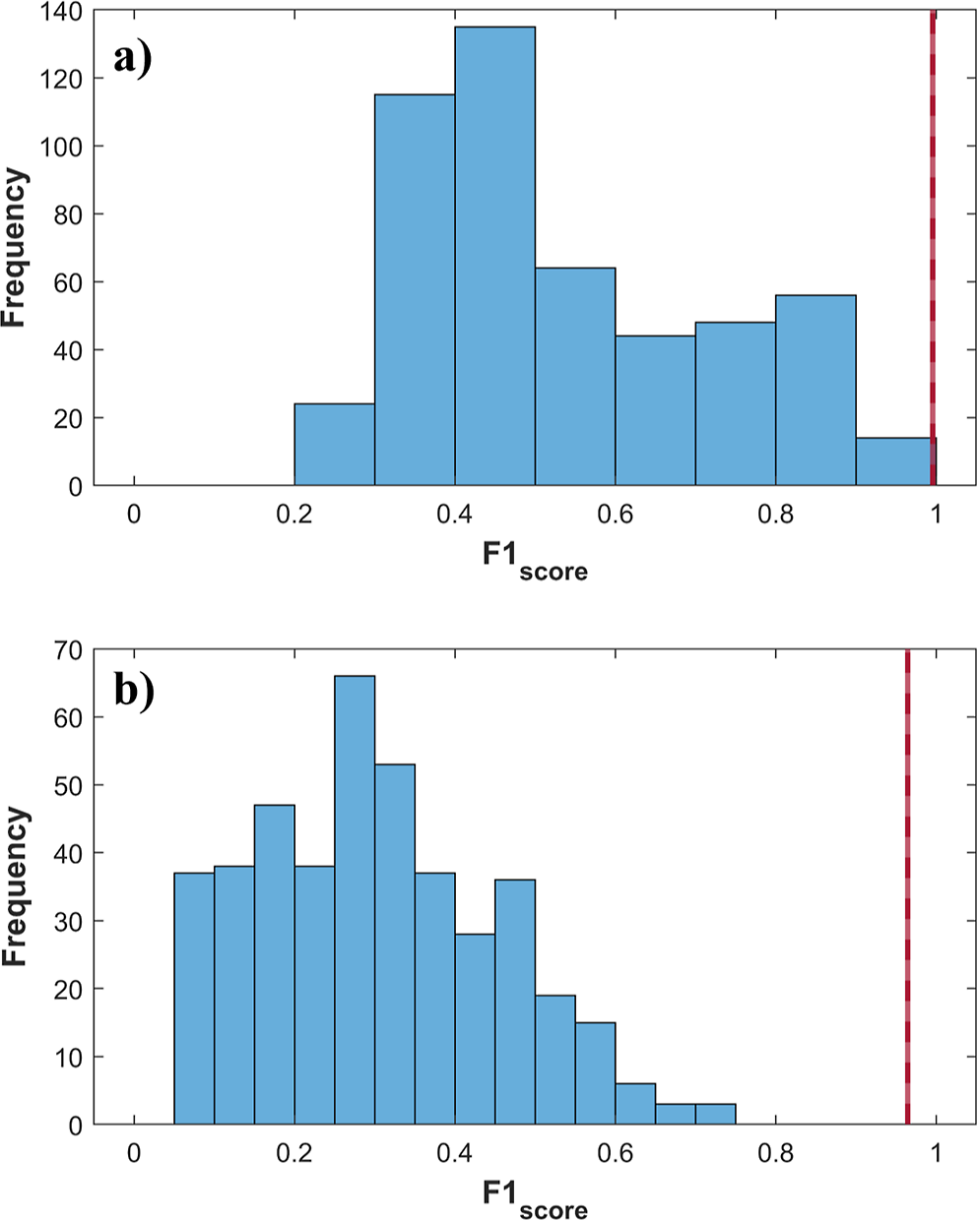
Permuted class vector for (a) training and (b) test sets in binary PLS-DA for the IO class. Blue bars indicate the results of the 500 permuted models and the red line indicates the results of original models.

## Results and Discussion

Tissue iron measure is clinically unfeasible; thus, given its limitations, an alternative approach is to estimate iron deposition in tissues using blood and multiomics techniques, such as mid-IR spectroscopy.^17^ To address this, artificial intelligence models were developed to determine whether IO is present, classify its severity, and predict iron concentrations in the blood and tissues. All results corroborate the innovative potential of developing a minimally invasive screening methodology for identifying and quantifying IO in one drop of blood, achieving excellent performance metrics using the combination of mid-IR spectroscopy and machine learning algorithms.

### Iron Overload

To confirm that iron-dextran administration induced the IO, iron content was measured in the blood, spleen, heart, liver, and kidney tissues (Table 1 and Figure S1). A significant increase was observed in the blood and all tissues in a dose-dependent manner. This result provided the necessary scenario for assessing the iron status using the proposed method. It enabled subsequent analyses since the clear differentiation of IO from controls and the identification of varying iron loading levels support its potential clinical utility.

### Binary Classification

The binary PLS-DA models were constructed using 7 ± 2 (mean ± standard deviation) of LV. The model was based on the spectral fingerprint region of mid-IR serum spectra. It was possible to distinguish the control and iron-loaded groups (250, 500, and 1000 mg·kg^–1^), highlighting the ability to identify IO. The binary PLS-DA model yielded excellent performance metrics for training and testing groups. Therefore, the models exhibited low FPR and FNR, reflecting the model’s excellent ability to discriminate interest classes (Table 2). Thus, 500 permutation tests were performed, and the results were compared with those of the original models to verify the prediction capability of the original models. Figure 2 shows that all models with the permuted class vector (blue bars) exhibited reliability rates significantly lower than those of the original models (red line). This data corroborated that the binary classification model is not overfitted or random and the metrics of the original model are accurate. Leal et al. (2021)^17^ employed FTIR spectroscopy to develop binary classification models to distinguish between two iron dosage levels in the blood (control vs 1000 mg·kg^–1^). Adel et al. (2021)^28^ proposed a methodology for treating IO in brain tissues, analyzed in 40 rat samples. FTIR spectroscopy was successfully employed to characterize specific spectral bands associated with IO, facilitating the identification of different functional groups. Likewise, Abd-Elghany and Mohamad (2021)^29^ utilized FTIR to evaluate the toxicity of iron oxide nanoparticles, aiming to explore their potential antitumor activity against Ehrlich carcinoma in mice.

**Table 2:** Performance Parameters (Mean ± Standard Deviation) of the Binary PLS-DA Models (LV = 7 ± 2)

parameter	train	test
ACC^a^	0.99 ± 0.01	0.95 ± 0.04
Sens.^b^	0.99 ± 0.01	0.94 ± 0.05
Spec.^c^	1.00 ± 0.01	0.95 ± 0.07
FPR^d^	0.00 ± 0.01	0.05 ± 0.07
FNR^e^	0.01 ± 0.01	0.06 ± 0.05

a Accuracy.

b Sensitivity.

c Specificity.

d False-positive rate.

e False-negative rate.

### Multiclass Classification

Next, we attempted to identify each IO class using spectral data. The performance parameters for the training and test sets are shown in Table 3. The multiclass model was constructed using LV = 11 ± 1. Like the binary class model, most samples were correctly classified into their respective class of interest (IO) in both the training and test groups. Misclassifications were primarily associated with the intermediate classes (250 and 500 mg·kg^–1^). While the spectral profiles of the control and 1000 mg·kg^–1^ groups showed pronounced spectral differences (Figure 3a), the spectral profiles of the 250 and 500 mg·kg^–1^ classes were more similar (Figure 3b). This spectral similarity may have limited the ability of FTIR spectroscopy to effectively discriminate between classes with higher iron concentrations. To improve the classification ability of the models, multiclass PLS-DA models with three classes were constructed by grouping the intermediate iron levels: class 1 (control), class 2 (250 and 500 mg·kg^–1^), and class 3 (1000 mg·kg^–1^). The results were more satisfactory when the intermediate iron levels were grouped together (250 and 500 mg·kg^–1^).

**Table 3:** Performance Parameters (Mean ± Standard Deviation) of the Multiclass PLS-DA (Four Class) Models (LV = 11 ± 1)

group	parameter	control	250 mg·kg^–1^	500 mg·kg^–1^	1000 mg·kg^–1^
train	ACC^a^	1.00 ± 0.03	0.99 ± 0.03	0.99 ± 0.03	1.00 ± 0.03
	Sens.^b^	1.00 ± 0.03	0.98 ± 0.04	0.99 ± 0.03	1.00 ± 0.03
	Spec.^c^	1.00 ± 0.03	1.00 ± 0.03	0.99 ± 0.03	1.00 ± 0.03
	FPR^d^	0.00 ± 0.00	0.00 ± 0.01	0.00 ± 0.01	0.00 ± 0.00
	FNR^e^	0.00 ± 0.00	0.01 ± 0.03	0.01 ± 0.03	0.00 ± 0.00
test	ACC^a^	0.97 ± 0.05	0.82 ± 0.09	0.81 ± 0.09	0.97 ± 0.05
	Sens.^b^	0.98 ± 0.10	0.62 ± 0.23	0.65 ± 0.23	0.95 ± 0.10
	Spec.^c^	0.97 ± 0.05	0.88 ± 0.09	0.88 ± 0.09	0.98 ± 0.05
	FPR^d^	0.03 ± 0.04	0.12 ± 0.09	0.12 ± 0.09	0.02 ± 0.04
	FNR^e^	0.02 ± 0.09	0.38 ± 0.23	0.35 ± 0.23	0.05 ± 0.10

a Accuracy.

b Sensitivity.

c Specificity.

d False-positive rate.

e False-negative rate.

**Figure 3:**
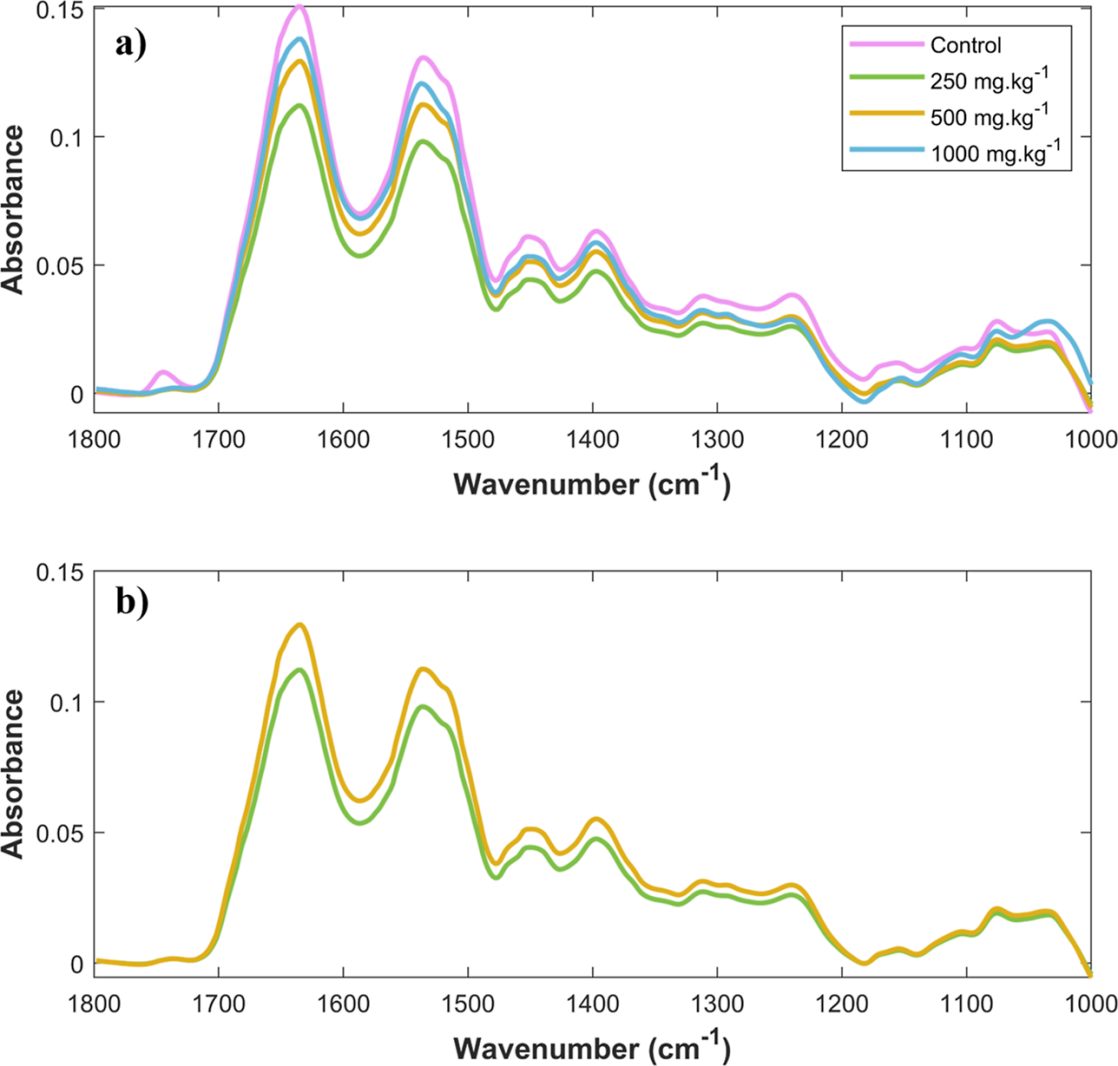
Average spectra of (a) control, 250 mg·kg^–1^, 500 mg·kg^–1^, and 1000 mg·kg^–1^, and (b) 250 mg·kg^–1^ and 500 mg·kg^–1^.

Once again, permuted models were constructed to verify the reliability of the classification of the PLS-DA model (Figure 4). The PLS-DA model showed significantly superior metrics (red line) compared to the 500 permuted models (blue bars). These results demonstrate that the multiclass model is not overfitting and that its prediction metric is reliable.

**Figure 4:**
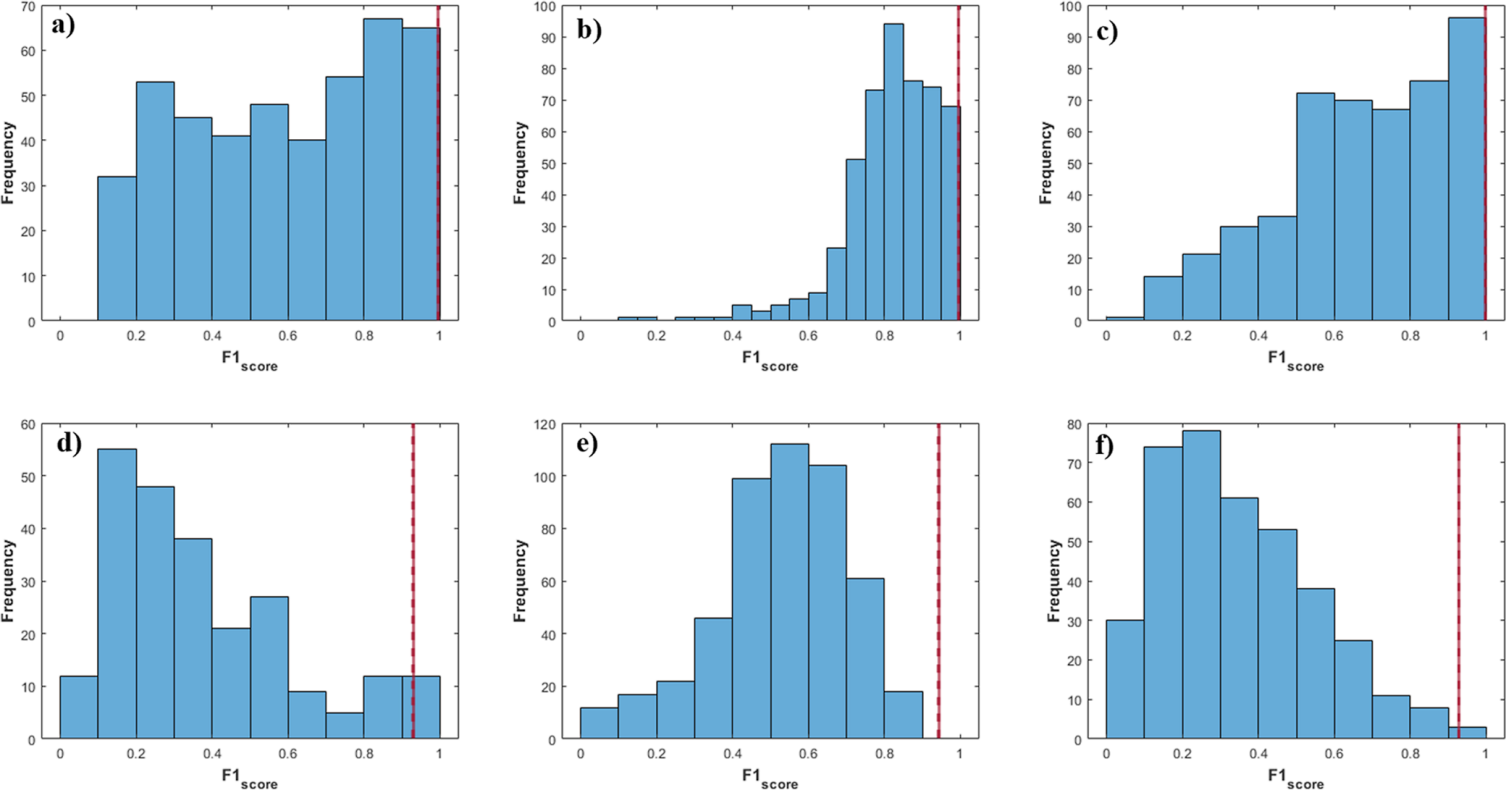
Permuted class vector in multiclass PLS-DA (three class) for the (a,d) control, (b,e) 250 and 500 mg·kg^–1^, and (c,f) 1000 mg·kg^–1^, for training (a–c) and test (d–f) sets. Blue bars indicate the results of the 500 permuted models and the red line indicates the results distribution for the original models.

To improve the classification ability of the models, multiclass PLS-DA models with three classes were constructed by grouping the intermediate iron levels: class 1 (control), class 2 (250 and 500 mg·kg^–1^), and class 3 (1000 mg·kg^–1^). The results (Table 4) were more satisfactory when the intermediate iron levels were grouped together (250 and 500 mg·kg^–1^). After grouping the intermediate classes, the model’s performance improved substantially, and the results demonstrated the promising potential of FTIR spectroscopy for iron identification, successfully classifying individuals into their respective groups. Finally, the permutation test was applied (Figure 5), highlighting the superior predictive capability of the model when grouping the intermediate classes, with the results from the original models significantly outperforming the permuted ones.

**Table 4:** Performance Parameters (Mean ± Standard Deviation) of the Multiclass PLS-DA (Three Class) Models (LV = 9 ± 2)

group	parameter	control	250 and 500 mg·kg^–1^	1000 mg·kg^–1^
train	ACC^a^	1.00 ± 0.03	1.00 ± 0.03	1.00 ± 0.03
	Sens.^b^	1.00 ± 0.03	0.99 ± 0.03	1.00 ± 0.03
	Spec.^c^	1.00 ± 0.03	1.00 ± 0.03	1.00 ± 0.03
	FPR^d^	0.00 ± 0.00	0.00 ± 0.00	0.00 ± 0.01
	FNR^e^	0.00 ± 0.00	0.01 ± 0.01	0.00 ± 0.00
test	ACC^a^	0.98 ± 0.04	0.92 ± 0.09	0.96 ± 0.05
	Sens.^b^	0.95 ± 0.13	0.92 ± 0.09	0.96 ± 0.09
	Spec.^c^	0.98 ± 0.04	0.96 ± 0.09	0.96 ± 0.06
	FPR^d^	0.02 ± 0.03	0.04 ± 0.07	0.04 ± 0.05
	FNR^e^	0.05 ± 0.12	0.08 ± 0.08	0.04 ± 0.09

a Accuracy.

b Sensitivity.

c Specificity.

d False-positive rate.

e False-negative rate.

**Figure 5:**
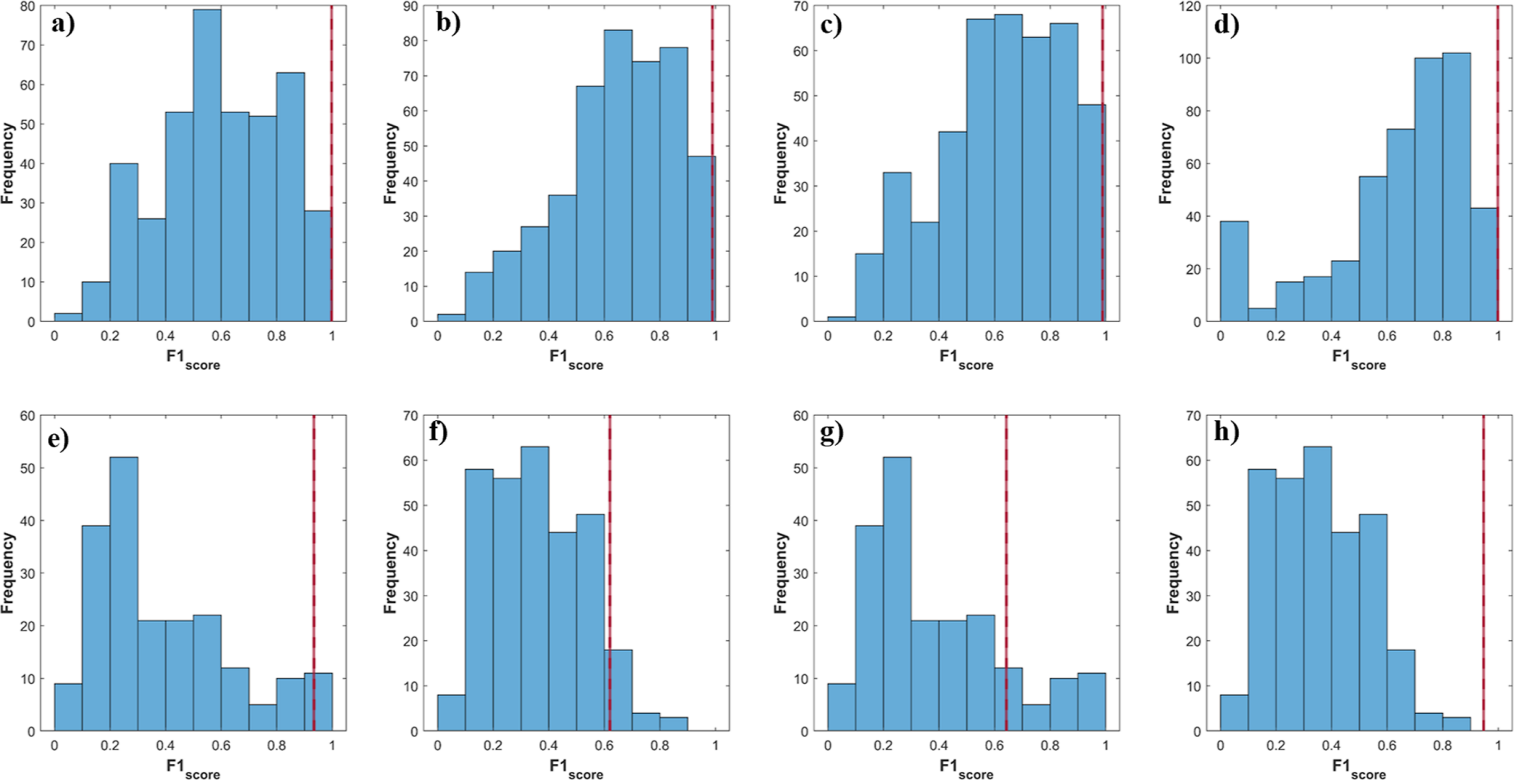
Permuted class vector in multiclass PLS-DA (four class) for the (a,e) control, (b,f) 250 mg·kg^–1^, (c,g) 500 mg·kg^–1^, and (d,h) 1000 mg·kg^–1^, for training (a–d) and test (e–h) sets. Blue bars indicate the results of the 500 permuted models and the red line indicates the results distribution for the original models.

### Regression

After being able to classify samples into different classes according to the level of iron loading through the serum spectrum, we applied regression models to predict the amount of iron accumulated in different organs. The evaluation parameters of the PLS model (Table 5) for both the calibration and test groups demonstrate that most tissues exhibited excellent linearity values (high values of *R* ^2^ for test (*R* ^2^p) and calibration (*R* ^2^c) sets) and low calibration and test errors. The LoD and LoQ were also satisfactory, particularly for higher iron concentrations, which were measured with greater confidence.

**Table 5:** Performance Parameters of the PLS Regression Model

model	blood	spleen	heart	liver	kidney
LV^a^	15	4	7	7	7
RMSEc^b^ (mg·kg^–1^)	478	2412	88	671	131
RMSEp^c^ (mg·kg^–1^)	1514	3937	127	867	243
*R* ^2^_c_ ^d^	1.00	0.82	0.99	0.92	0.98
*R* ^2^_p_ ^e^	0.95	0.85	0.98	0.91	0.90
LoD^f^ (mg·kg^–1^)	705	472	49	362	69
LoQ^g^ (mg·kg^–1^)	2351	1573	165	1208	231

a Latent variables.

b Root-mean-square error of calibration.

c Root-mean-square error of test.

d Linearity of calibration.

e Linearity of test.

f Limit of detection.

g Limit of quantification.

Figure 6 shows that it was possible to predict iron levels in serum (Figure 6a) and organs studied (spleen, Figure 6b; heart, Figure 6c; liver, Figure 6d; and kidney, Figure 6e) using only a single drop of blood, eliminating the need for invasive biopsies. A real correlation was observed between the chemical information and the measured iron values for each target property (blood serum and tissues). Notably, the model to spleen exhibited lower linearity between experimental and predicted values than the model to blood, heart, liver, and kidney, indicating reliable predictions (Figure 6).

**Figure 6:**
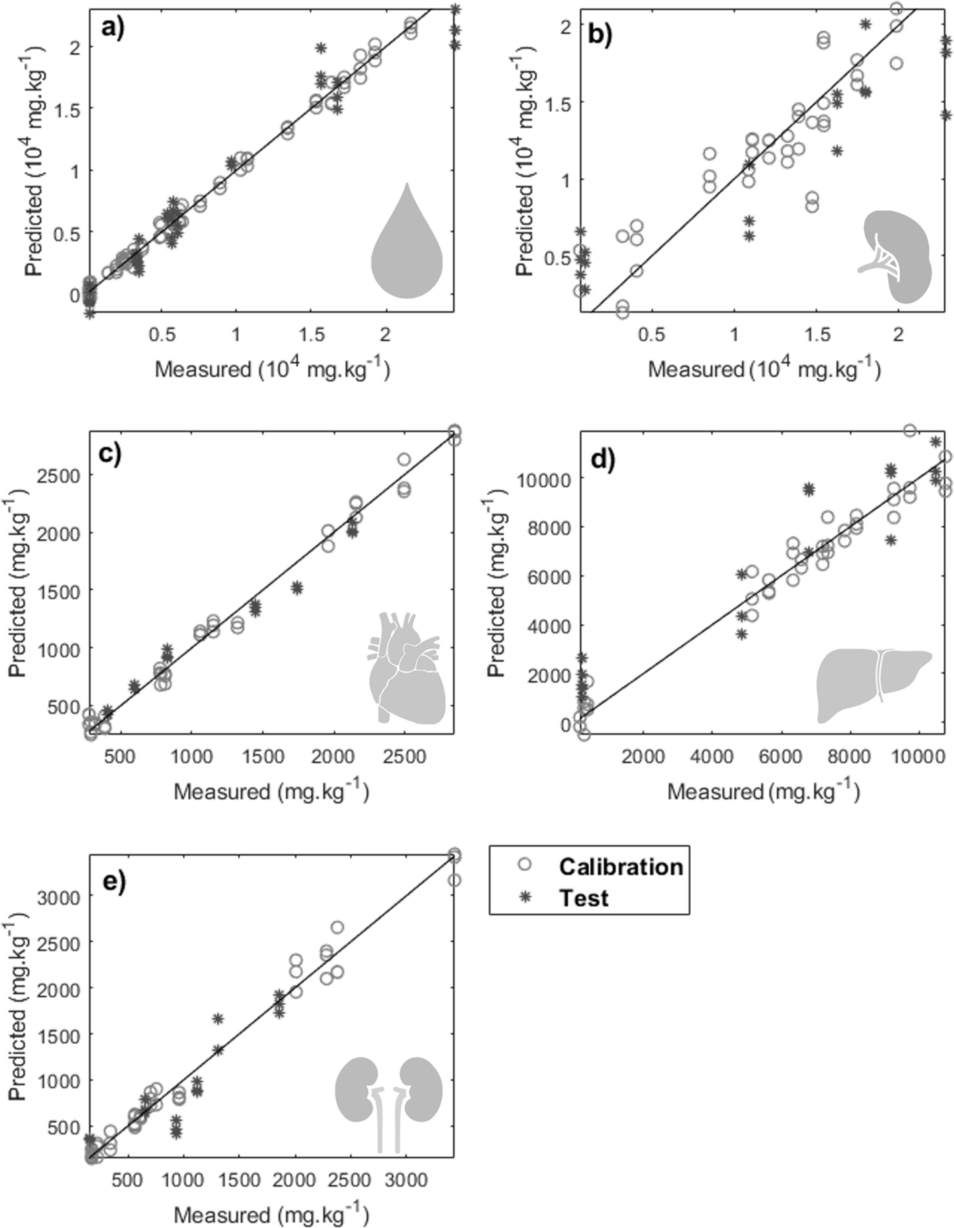
PLS regression of iron dosage measurements in (a) blood, (b) spleen, (c) heart, (d) liver, and (e) kidney.

Permutation tests were also conducted on the regression vector to analyze errors in the calibration and test groups (Figure 7). All permuted models exhibited significantly higher errors for both calibration (Figure 7a–e) and validation (test) groups (Figure 7f–j). The original models demonstrate statistical significance and establish a cause-effect relation in quantifying iron levels in the blood (Figure 7a,f), spleen (Figure 7b,g), heart (Figure 7c,h), liver (Figure 7d,i), and kidney (Figure 7e,j). Therefore, these findings support the potential of the proposed models for estimating iron accumulation in organs with robust performance metrics, using only one drop of blood, without requiring invasive biopsies. It should make this method viable for large-scale screening of patients suspected of iron intoxication.

**Figure 7:**
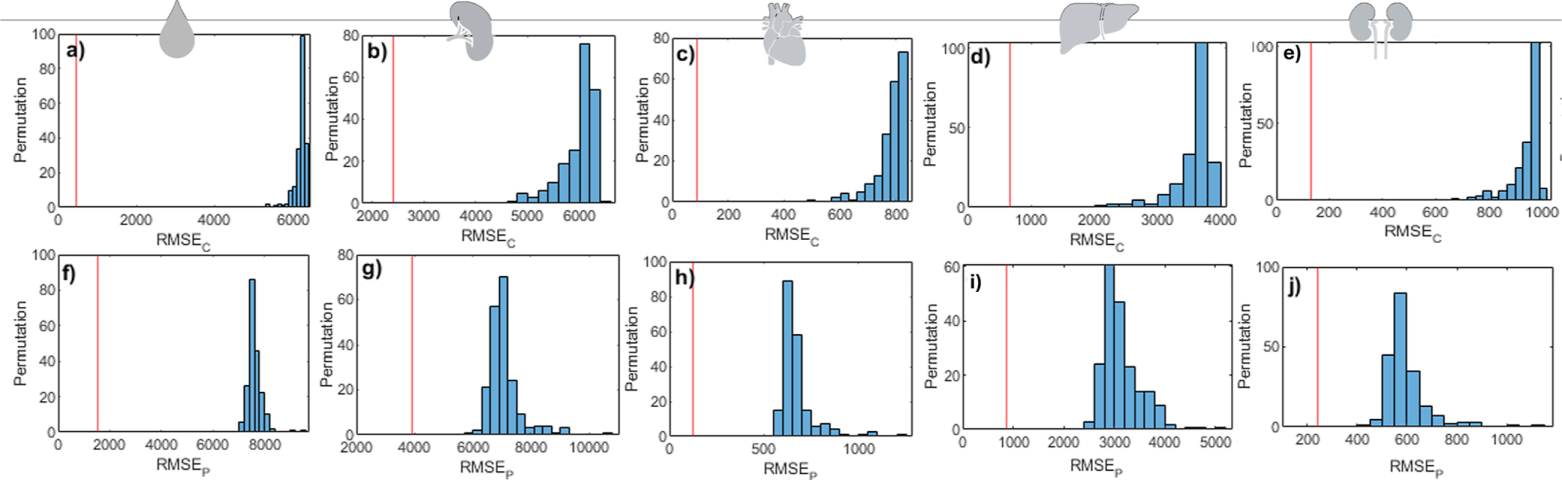
Permuted class vector in PLS regression model or quantifying iron measures in (a,f) blood, (b,g) spleen, (c,h) heart, (d,i) liver, and (e,j) kidney for samples in the (a–e) calibration group and (f–j) test sets. Blue and red bars indicate the results for the permuted and original models, respectively.

### Molecular Correlation

Raw spectra for all classes were constructed to identify the cause-effect relation in the spectral chemical information to iron measurements (Figure 8a), alongside preprocessed spectra emphasizing the most important variables for each constructed model. These include binary classification (Figure 8b), multiclass classification (Figure 8c), and regression models for quantifying iron in blood (Figure 8d), spleen (Figure 8e), heart (Figure 8f), liver (Figure 8g), and kidney (Figure 8h). The critical spectral regions identified for the classification and regression models were 1125–1250 cm^–1^, 1280–1330 cm^–1^, and 1350–1450 cm^–1^. The first region corresponds to C–O stretching vibrations, C–C bonds, and hydrogen-bonded C–OH groups, potentially associated with carbohydrates, phospholipids, polysaccharides, pectin, and/or lactate.^30–34^ The second region shows C–O signals that may indicate saccharides, glucose, lactate, and glycerol.^28,29,31^ The third region reflects the presence of amino acids and proteins, with COO^–^ stretching, NH bending, and CN stretching in amide I, symmetric COO^–^ vibration in amide III, and C–C bonds.^28,30–35^ Mid-IR spectroscopy does not exhibit specific absorption regions for iron. However, these spectral changes may reflect adaptations associated with iron-induced dysregulation. Alterations in biomolecules could reflect systemic stress responses and may result from free radicals generated by the iron-induced Fenton reaction.^17,36,37^ Alterations in these regions reflect tissue metabolic changes resulting from iron-induced oxidative stress (ROS) and low hepcidin levels.^17^ Such oxidative damage can lead to hemolysis, changes in the circulation of albumin, citrate, acetate, malate, and phosphate, as well as conditions like anemia and hypoxia.^17,38–40^

**Figure 8:**
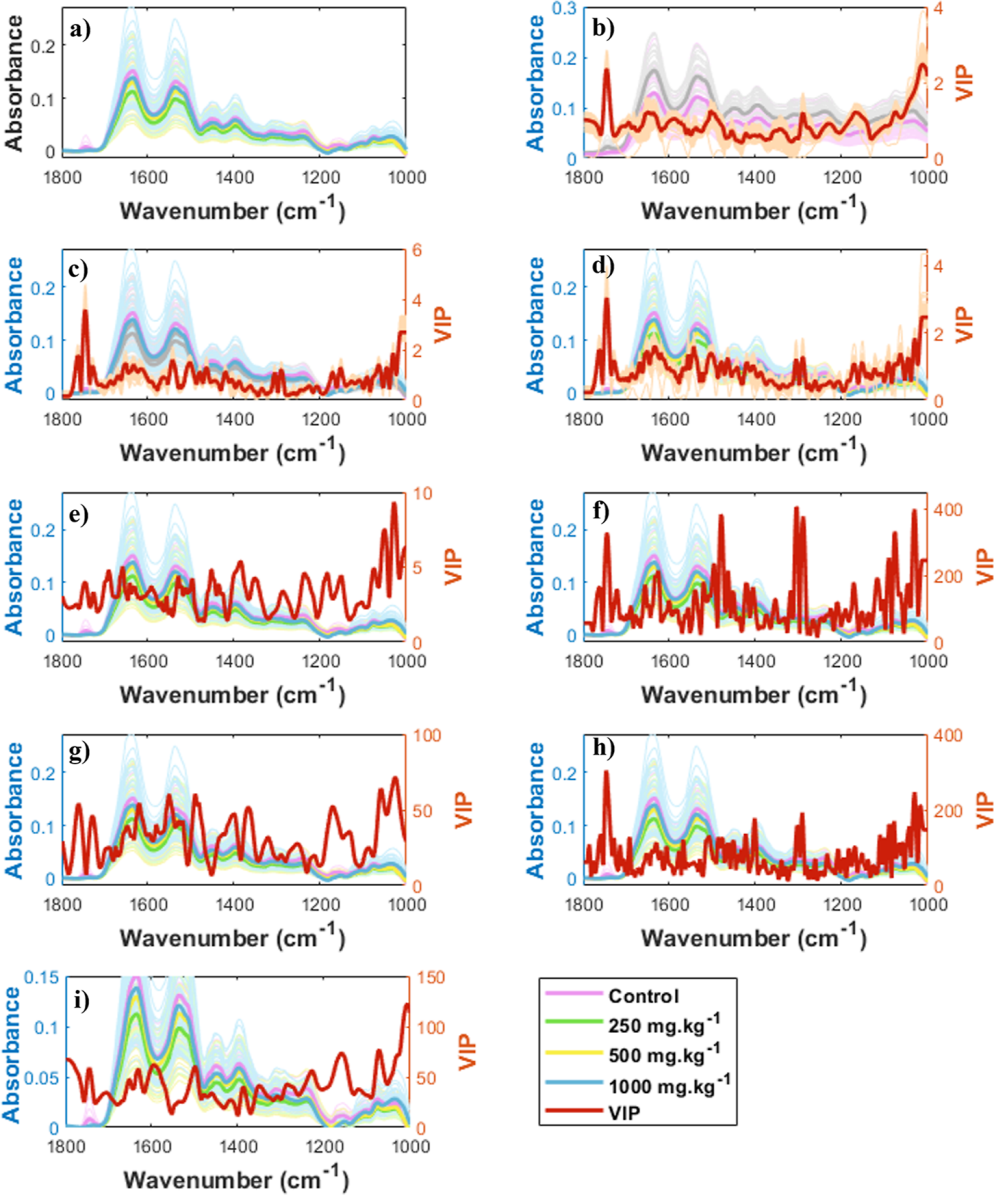
(a) Original spectra, preprocessed spectra, and VIPs for the PLS-DA classification model: (b) binary, (c) multiclass, and PLS regression models for (d) blood, (e) spleen, (f) heart, (g) liver, and (h) kidney.

The most susceptible tissues to IO-related damage are those characterized by substantial iron accumulation and elevated metabolic activity (e.g., liver and cardiovascular system), where ROS production and subsequent oxidative stress play a key role in the pathological process.^41–43^

Notably, regression models for the spleen (Figure 8e) and liver (Figure 8g) share similar critical spectral regions as well as for the heart (Figure 8f) and kidney (Figure 8h). These similarities can be associated with their functional interconnection within the reticuloendothelial system^44–46^ and cardiorenal system.^47–49^ However, targeted metabolomic or proteomic analyses of these organs are necessary to confirm the distinction between organ-specific and systemic effects.

## Conclusions

Using mid-IR spectroscopy associated with chemometric methods, an innovative and promising screening methodology was developed to identify iron loading at various levels and quantify iron in the blood and multiple tissues (spleen, heart, liver, and kidney) without requiring biopsies using mid-IR spectroscopy associated with chemometric methods.

Binary classification models (indicating the presence or absence of IO) and multiclass models (control, 250, 500, and 1000 mg·kg^–1^) demonstrate exceptional performance, achieving 100% spec., sens., and ACC, with no occurrences of false positives or negatives, for both the training and external validation groups. Regression models for quantifying iron in the blood, spleen, heart, liver, and kidney show excellent linearity and low associated errors in calibration and external validation groups.

The spectral information from the original models provides accurate chemical insights, correlating with oxidative stress caused by IO. Furthermore, interrelated patterns were observed between the spleen and liver as well as the heart and kidney, underscoring how dysfunction in one organ can directly influence the performance of the other.

Our proposed method could offer several key advantages: its minimally invasive nature eliminates the need for biopsies, reducing patient discomfort and associated risks; it is readily adaptable to multiple point-of-care settings, enabling rapid and decentralized testing; it is scalable for large-scale screening programs, facilitating broader population coverage and earlier detection; and it promises to be a cost-effective alternative to existing, more complex diagnostic procedures. In an era of escalating healthcare expenditures, cost-benefit considerations are paramount to public health decision-making. Therefore, this research shows promise for future studies of human screening and encourages further clinical research to evaluate its effectiveness.

## Supplementary Material



## Notes

Number of LV *versus* RMSE from PLS cross-validation for iron quantification in blood, spleen, heart, liver, and kidney; iron measurement in blood, spleen, heart, liver, and kidney; and performance parameters of the binary and multiclass PLS-DA models using different pre-treatments (PDF)
