# Supplementary material for: One-Drop Serum Screening Test to Monitor Tissue Iron Accumulation
Source: Anal Chem. 2025 May 30;97(22):11598–608. doi: 10.1021/acs.analchem.5c00778 (PMC12163878; doi:10.1021/acs.analchem.5c00778)
Supplement: Supplementary file 1 [file ac5c00778_si_001.pdf]

Supporting Information

One-drop serum Screening Test to Monitor Tissue Iron Accumulation

Gabriely S. Folli[a,b], Anne Louise S. Torres[b], Matthews Martins[b], Luiz Ricardo Rodrigues Silva[b], Vinícius Bermond Marques[b], Maria Tereza Carneiro[a], Larissa Dias Roriz[a], Leonardo dos Santos[b], Wanderson Romão[c], Francis L. Martin[d], Paulo R. Filgueiras[a], Valério G. Barauna[b]*

[a] Departamento of Chemistry, Federal University of Espírito Santo (UFES), Av. Fernando Ferrari, 514, Vitória, Espírito Santo, Zip Code: 29075-910, Brazil.

[b] Department of Physiological Sciences, Federal University of Espírito Santo (UFES), Av. Marechal Campos, 1468, Maruípe, Vitória, Espírito Santo 29040-090, Brazil

[c] Federal Institute of Education, Science, and Technology of Espírito Santo, Av. Ministro Salgado Filho, 1000, Vila Velha, Espírito Santo, Zip Code: 29106-010, Brazil.

[d] Department of Cellular Pathology, Blackpool Teaching Hospitals NHS Foundation Trust, Whinney Heys Road, Blackpool, FY3 8NR, UK.

*Corresponding author. E-mail addresses: valerio.barauna@ufes.br or barauna2@gmail.com

Table of Content

Figure S1. Number of LV *versus* RMSE from PLS cross-validation for iron quantification in (a) blood, (b) spleen, (c) heart, (d) liver, and (e) kidney……………………………………………………..…………………S3

Figure S2. Iron measurement in (a) blood, (b) spleen, (c) heart, (d) liver, and (e) kidney. Data represents the mean ± standard deviation of the mean. *p<0.05 vs. control..………………………………………………S4

Table S1. Performance parameters of the binary PLS-DA model using different pre-treatments for class 1 (control) ……………………………………………………………………..…………..……..…...…S5

Table S2. Performance parameters of the multiclass PLS-DA model using different pre-treatments for class 1 (control) ………………………………………………………………………………..………...……S9

Table S3. Performance parameters of the multiclass PLS-DA model using different pre-treatments for class 2 (250 mg.kg^{-1}) ………………………………………………………………………………...………S13

Table S4. Performance parameters of the multiclass PLS-DA model using different pre-treatments for class 3 (500 mg.kg^{-1}) ……………………………………………………………………….………...………S17

Table S5. Performance parameters of the multiclass PLS-DA model using different pre-treatments for class 4 (1000 mg.kg^{-1}) ……………………………………………………………………………..……………S21

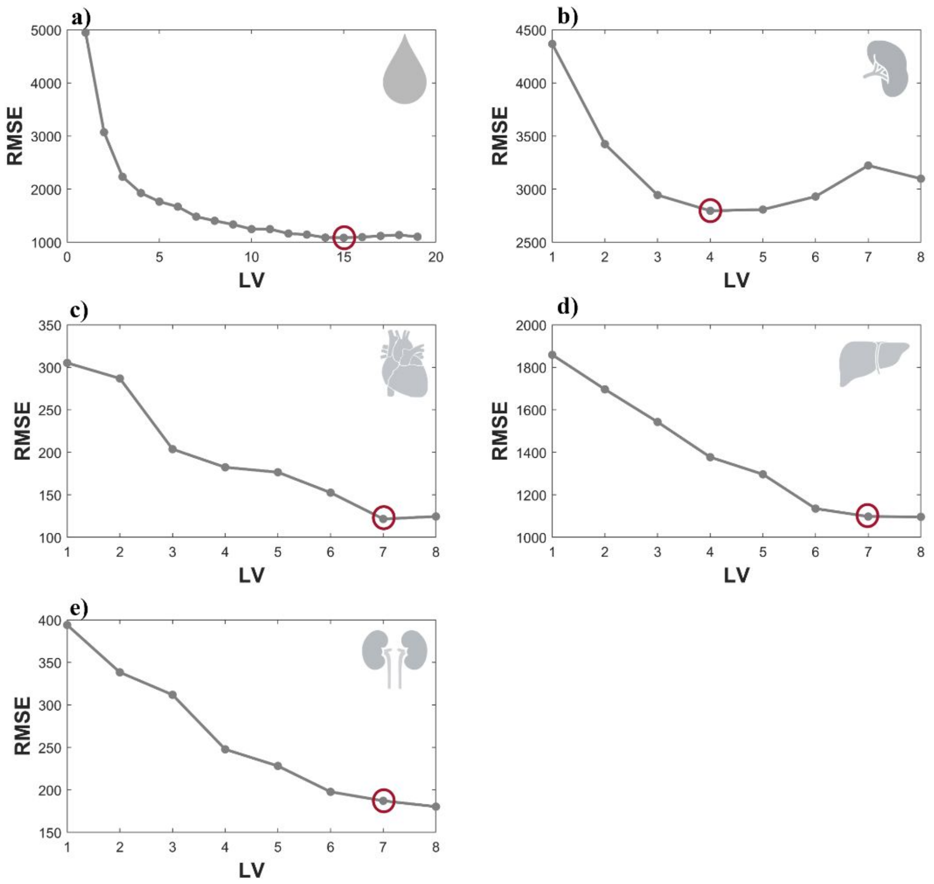

Figure S1. Number of LV *versus* RMSE from PLS cross-validation for iron quantification in (a) blood, (b) spleen, (c) heart, (d) liver, and (e) kidney.

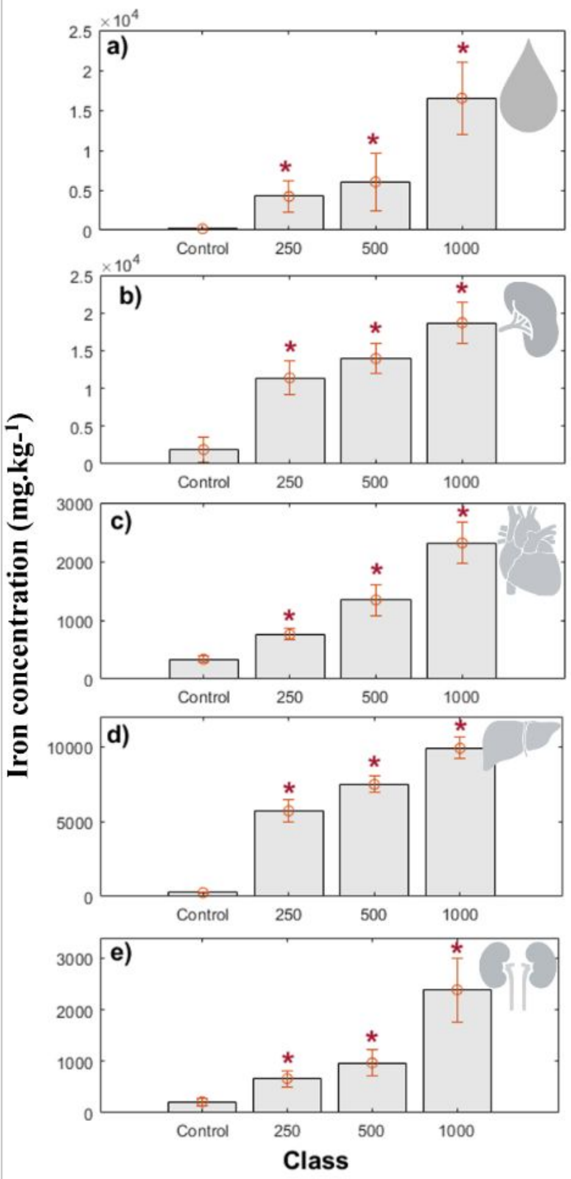

Figure S2. Iron measurement in (a) blood, (b) spleen, (c) heart, (d) liver, and (e) kidney. Data represents the mean ± standard deviation of the mean. *p<0.05 vs. control.

Table S1. Performance parameters of the binary PLS-DA model using different pre-pretreatment for class 1 (control).

Pre-pretreatment			Train					Test				
**airPLS**	**Pretrat2**	**Pretrat3**	**ACC[1]**	**Sens.[2]**	**Spec[3]**	**FPR[4]**	**FNR[5]**	**ACC[1]**	**Sens.[2]**	**Spec[3]**	**FPR[4]**	**FNR[5]**
no	svn	none	1,0	1,0	1,0	0,0	0,0	1,0	1,0	1,0	0,0	0,0
no	snv	derivate (window =7; polynomial = 2, degree = 0)	1,0	1,0	1,0	0,0	0,0	1,0	1,0	1,0	0,0	0,0
yes	snv	none	1,0	1,0	1,0	0,0	0,0	1,0	1,0	1,0	0,0	0,0
yes	norm	none	1,0	1,0	1,0	0,0	0,0	1,0	1,0	1,0	0,0	0,0
yes	derivate (window =15; polynomial = 2, degree = 1)	none	1,0	1,0	1,0	0,0	0,0	1,0	1,0	1,0	0,0	0,0
yes	derivate (window =15; polynomial = 2, degree = 0)	none	1,0	1,0	1,0	0,0	0,0	1,0	1,0	1,0	0,0	0,0
yes	derivate (window =21; polynomial = 2, degree = 0)	none	1,0	1,0	1,0	0,0	0,0	1,0	1,0	1,0	0,0	0,0
yes	snv	derivate (window =15; polynomial = 2, degree = 1)	1,0	1,0	1,0	0,0	0,0	1,0	1,0	1,0	0,0	0,0
yes	snv	derivate (window =7; polynomial = 2, degree = 0)	1,0	1,0	1,0	0,0	0,0	1,0	1,0	1,0	0,0	0,0
yes	snv	derivate (window =15; polynomial = 2, degree = 0)	1,0	1,0	1,0	0,0	0,0	1,0	1,0	1,0	0,0	0,0
yes	snv	derivate (window =21; polynomial = 2, degree = 0)	1,0	1,0	1,0	0,0	0,0	1,0	1,0	1,0	0,0	0,0
yes	msc	derivate (window =7; polynomial = 2, degree = 0)	1,0	1,0	1,0	0,0	0,0	1,0	1,0	1,0	0,0	0,0
yes	msc	derivate (window =15; polynomial = 2, degree = 0)	1,0	1,0	1,0	0,0	0,0	1,0	1,0	1,0	0,0	0,0
yes	msc	derivate (window =21; polynomial = 2, degree = 0)	1,0	1,0	1,0	0,0	0,0	1,0	1,0	1,0	0,0	0,0
no	snv	derivate (window =15; polynomial = 2, degree = 0)	1,0	1,0	1,0	0,0	0,0	1,0	1,0	1,0	0,0	0,0
yes	snv	derivate (window =21; polynomial = 2, degree = 1)	1,0	1,0	1,0	0,0	0,0	1,0	1,0	1,0	0,0	0,0
yes	msc	derivate (window =15; polynomial = 2, degree = 1)	1,0	1,0	1,0	0,0	0,0	1,0	1,0	1,0	0,0	0,0
yes	msc	derivate (window =21; polynomial = 2, degree = 1)	1,0	1,0	1,0	0,0	0,0	1,0	1,0	1,0	0,0	0,0

Pre-pretreatment			Train					Test				
**airPLS**	**Pretrat2**	**Pretrat3**	**ACC[1]**	**Sens.[2]**	**Spec[3]**	**FPR[4]**	**FNR[5]**	**ACC[1]**	**Sens.[2]**	**Spec[3]**	**FPR[4]**	**FNR[5]**
no	msc	none	1,0	1,0	1,0	0,0	0,0	1,0	0,9	1,0	0,0	0,1
no	norm	none	1,0	1,0	1,0	0,0	0,0	1,0	0,9	1,0	0,0	0,1
no	derivate (window =9; polynomial = 2, degree = 2)	none	1,0	1,0	1,0	0,0	0,0	1,0	0,9	1,0	0,0	0,1
no	snv	derivate (window =15; polynomial = 2, degree = 2)	1,0	1,0	1,0	0,0	0,0	1,0	0,9	1,0	0,0	0,1
no	msc	derivate (window =15; polynomial = 2, degree = 2)	1,0	1,0	1,0	0,0	0,0	1,0	0,9	1,0	0,0	0,1
no	msc	derivate (window =7; polynomial = 2, degree = 0)	1,0	1,0	1,0	0,0	0,0	1,0	0,9	1,0	0,0	0,1
yes	none	none	1,0	1,0	1,0	0,0	0,0	1,0	0,9	1,0	0,0	0,1
yes	msc	none	1,0	1,0	1,0	0,0	0,0	1,0	0,9	1,0	0,0	0,1
yes	derivate (window =9; polynomial = 2, degree = 1)	none	1,0	1,0	1,0	0,0	0,0	1,0	0,9	1,0	0,0	0,1
yes	derivate (window =9; polynomial = 2, degree = 2)	none	1,0	1,0	1,0	0,0	0,0	1,0	0,9	1,0	0,0	0,1
yes	derivate (window =7; polynomial = 2, degree = 0)	none	1,0	1,0	1,0	0,0	0,0	1,0	0,9	1,0	0,0	0,1
yes	snv	derivate (window =9; polynomial = 2, degree = 1)	1,0	1,0	1,0	0,0	0,0	1,0	1,0	0,9	0,1	0,0
yes	msc	derivate (window =9; polynomial = 2, degree = 1)	1,0	1,0	1,0	0,0	0,0	1,0	1,0	0,9	0,1	0,0
no	none	none	1,0	1,0	1,0	0,0	0,0	1,0	0,9	1,0	0,0	0,1
no	snv	derivate (window =9; polynomial = 2, degree = 2)	1,0	1,0	1,0	0,0	0,0	1,0	0,9	1,0	0,0	0,1
no	msc	derivate (window =9; polynomial = 2, degree = 2)	1,0	1,0	1,0	0,0	0,0	1,0	0,9	1,0	0,0	0,1
no	msc	derivate (window =15; polynomial = 2, degree = 0)	1,0	1,0	1,0	0,0	0,0	1,0	0,9	1,0	0,0	0,1
yes	snv	derivate (window =9; polynomial = 2, degree = 2)	1,0	1,0	1,0	0,0	0,0	1,0	0,9	1,0	0,0	0,1
yes	msc	derivate (window =9; polynomial = 2, degree = 2)	1,0	1,0	1,0	0,0	0,0	1,0	0,9	1,0	0,0	0,1

Pre-pretreatment			Train					Test				
**airPLS**	**Pretrat2**	**Pretrat3**	**ACC[1]**	**Sens.[2]**	**Spec[3]**	**FPR[4]**	**FNR[5]**	**ACC[1]**	**Sens.[2]**	**Spec[3]**	**FPR[4]**	**FNR[5]**
no	derivate (window =15; polynomial = 2, degree = 2)	none	1,0	1,0	1,0	0,0	0,0	1,0	0,8	1,0	0,0	0,2
yes	derivate (window =15; polynomial = 2, degree = 2)	none	1,0	1,0	1,0	0,0	0,0	1,0	0,8	1,0	0,0	0,2
yes	snv	derivate (window =15; polynomial = 2, degree = 2)	1,0	1,0	1,0	0,0	0,0	1,0	0,8	1,0	0,0	0,2
yes	msc	derivate (window =15; polynomial = 2, degree = 2)	1,0	1,0	1,0	0,0	0,0	1,0	0,8	1,0	0,0	0,2
no	snv	derivate (window =15; polynomial = 2, degree = 1)	1,0	1,0	1,0	0,0	0,0	0,9	1,0	0,9	0,1	0,0
no	snv	derivate (window =21; polynomial = 2, degree = 0)	1,0	1,0	1,0	0,0	0,0	0,9	1,0	0,9	0,1	0,0
no	msc	derivate (window =15; polynomial = 2, degree = 1)	1,0	1,0	1,0	0,0	0,0	0,9	1,0	0,9	0,1	0,0
no	derivate (window =9; polynomial = 2, degree = 1)	none	1,0	1,0	1,0	0,0	0,0	0,9	0,9	0,9	0,1	0,1
no	derivate (window =7; polynomial = 2, degree = 0)	none	1,0	1,0	1,0	0,0	0,0	0,9	0,9	0,9	0,1	0,1
no	msc	derivate (window =9; polynomial = 2, degree = 1)	1,0	1,0	1,0	0,0	0,0	0,9	0,9	0,9	0,1	0,1
no	msc	derivate (window =21; polynomial = 2, degree = 0)	1,0	1,0	1,0	0,0	0,0	0,9	0,9	0,9	0,1	0,1
yes	derivate (window =21; polynomial = 2, degree = 1)	none	1,0	1,0	1,0	0,0	0,0	0,9	0,9	0,9	0,1	0,1
no	snv	derivate (window =9; polynomial = 2, degree = 1)	1,0	1,0	1,0	0,0	0,0	0,9	0,9	0,9	0,1	0,1
no	snv	derivate (window =21; polynomial = 2, degree = 2)	1,0	1,0	1,0	0,0	0,0	0,9	0,8	0,9	0,1	0,2
no	msc	derivate (window =21; polynomial = 2, degree = 2)	1,0	1,0	1,0	0,0	0,0	0,9	0,8	0,9	0,1	0,2
no	derivate (window =21; polynomial = 2, degree = 0)	none	1,0	1,0	1,0	0,0	0,0	0,9	0,9	0,9	0,1	0,1
no	msc	derivate (window =21; polynomial = 2, degree = 1)	1,0	1,0	1,0	0,0	0,0	0,8	1,0	0,8	0,2	0,0

Pre-pretreatment			Train					Test				
**airPLS**	**Pretrat2**	**Pretrat3**	**ACC[1]**	**Sens.[2]**	**Spec[3]**	**FPR[4]**	**FNR[5]**	**ACC[1]**	**Sens.[2]**	**Spec[3]**	**FPR[4]**	**FNR[5]**
yes	snv	derivate (window =21; polynomial = 2, degree = 2)	1,0	1,0	1,0	0,0	0,0	0,9	0,7	0,9	0,1	0,3
yes	msc	derivate (window =21; polynomial = 2, degree = 2)	1,0	1,0	1,0	0,0	0,0	0,9	0,7	0,9	0,1	0,3
no	derivate (window =15; polynomial = 2, degree = 0)	none	1,0	1,0	1,0	0,0	0,0	0,8	0,9	0,8	0,2	0,1
no	derivate (window =21; polynomial = 2, degree = 1)	none	1,0	1,0	1,0	0,0	0,0	0,8	0,9	0,8	0,2	0,1
no	snv	derivate (window =21; polynomial = 2, degree = 1)	1,0	1,0	1,0	0,0	0,0	0,8	1,0	0,8	0,3	0,0
no	derivate (window =15; polynomial = 2, degree = 1)	none	1,0	1,0	1,0	0,0	0,0	0,8	0,9	0,8	0,2	0,1
no	derivate (window =21; polynomial = 2, degree = 2)	none	1,0	1,0	1,0	0,0	0,0	0,8	0,6	0,8	0,2	0,4
yes	derivate (window =21; polynomial = 2, degree = 2)	none	1,0	1,0	1,0	0,0	0,0	0,8	0,6	0,8	0,2	0,4

[1]Accuracy; [2]Sensitivity; [3]Specificity; [4]False Positive Rate ; [5]False Negative Rate.

Table S2. Performance parameters of the multiclass PLS-DA model using different pre-treatments for class 1 (control).

Pre-pretreatment			Train					Test				
**airPLS**	**Pretrat2**	**Pretrat3**	**ACC[1]**	**Sens.[2]**	**Spec[3]**	**FPR[4]**	**FNR[5]**	**ACC[1]**	**Sens.[2]**	**Spec[3]**	**FPR[4]**	**FNR[5]**
no	svn	none	1,0	1,0	1,0	0,0	0,0	1,0	1,0	1,0	0,0	0,0
no	snv	derivate (window =7; polynomial = 2, degree = 0)	1,0	1,0	1,0	0,0	0,0	1,0	1,0	1,0	0,0	0,0
yes	snv	none	1,0	1,0	1,0	0,0	0,0	1,0	1,0	1,0	0,0	0,0
yes	norm	none	1,0	1,0	1,0	0,0	0,0	1,0	1,0	1,0	0,0	0,0
yes	derivate (window =15; polynomial = 2, degree = 1)	none	1,0	1,0	1,0	0,0	0,0	1,0	1,0	1,0	0,0	0,0
yes	derivate (window =15; polynomial = 2, degree = 0)	none	1,0	1,0	1,0	0,0	0,0	1,0	1,0	1,0	0,0	0,0
yes	derivate (window =21; polynomial = 2, degree = 0)	none	1,0	1,0	1,0	0,0	0,0	1,0	1,0	1,0	0,0	0,0
yes	snv	derivate (window =15; polynomial = 2, degree = 1)	1,0	1,0	1,0	0,0	0,0	1,0	1,0	1,0	0,0	0,0
yes	snv	derivate (window =7; polynomial = 2, degree = 0)	1,0	1,0	1,0	0,0	0,0	1,0	1,0	1,0	0,0	0,0
yes	snv	derivate (window =15; polynomial = 2, degree = 0)	1,0	1,0	1,0	0,0	0,0	1,0	1,0	1,0	0,0	0,0
yes	snv	derivate (window =21; polynomial = 2, degree = 0)	1,0	1,0	1,0	0,0	0,0	1,0	1,0	1,0	0,0	0,0
yes	msc	derivate (window =7; polynomial = 2, degree = 0)	1,0	1,0	1,0	0,0	0,0	1,0	1,0	1,0	0,0	0,0
yes	msc	derivate (window =15; polynomial = 2, degree = 0)	1,0	1,0	1,0	0,0	0,0	1,0	1,0	1,0	0,0	0,0
yes	msc	derivate (window =21; polynomial = 2, degree = 0)	1,0	1,0	1,0	0,0	0,0	1,0	1,0	1,0	0,0	0,0
no	snv	derivate (window =15; polynomial = 2, degree = 0)	1,0	1,0	1,0	0,0	0,0	1,0	1,0	1,0	0,0	0,0
yes	snv	derivate (window =21; polynomial = 2, degree = 1)	1,0	1,0	1,0	0,0	0,0	1,0	1,0	1,0	0,0	0,0
yes	msc	derivate (window =15; polynomial = 2, degree = 1)	1,0	1,0	1,0	0,0	0,0	1,0	1,0	1,0	0,0	0,0
yes	msc	derivate (window =21; polynomial = 2, degree = 1)	1,0	1,0	1,0	0,0	0,0	1,0	1,0	1,0	0,0	0,0

Pre-pretreatment			Train					Test				
airPLS	Pretrat2	Pretrat3	ACC[1]	Sens.[2]	Spec[3]	FPR[4]	FNR[5]	ACC[1]	Sens.[2]	Spec[3]	FPR[4]	FNR[5]
no	msc	none	1,0	1,0	1,0	0,0	0,0	1,0	1,0	1,0	0,0	0,0
no	norm	none	1,0	1,0	1,0	0,0	0,0	1,0	1,0	1,0	0,0	0,0
no	derivate (window =9; polynomial = 2, degree = 2)	none	1,0	1,0	1,0	0,0	0,0	1,0	1,0	1,0	0,0	0,0
no	snv	derivate (window =15; polynomial = 2, degree = 2)	1,0	1,0	1,0	0,0	0,0	1,0	1,0	1,0	0,0	0,0
no	msc	derivate (window =15; polynomial = 2, degree = 2)	1,0	1,0	1,0	0,0	0,0	1,0	1,0	1,0	0,0	0,0
no	msc	derivate (window =7; polynomial = 2, degree = 0)	1,0	1,0	1,0	0,0	0,0	1,0	1,0	1,0	0,0	0,0
yes	none	none	1,0	1,0	1,0	0,0	0,0	1,0	1,0	1,0	0,0	0,0
yes	msc	none	1,0	1,0	1,0	0,0	0,0	1,0	1,0	1,0	0,0	0,0
yes	derivate (window =9; polynomial = 2, degree = 1)	none	1,0	1,0	1,0	0,0	0,0	1,0	1,0	1,0	0,0	0,0
yes	derivate (window =9; polynomial = 2, degree = 2)	none	1,0	1,0	1,0	0,0	0,0	1,0	1,0	1,0	0,0	0,0
yes	derivate (window =7; polynomial = 2, degree = 0)	none	1,0	1,0	1,0	0,0	0,0	1,0	1,0	1,0	0,0	0,0
yes	snv	derivate (window =9; polynomial = 2, degree = 1)	1,0	1,0	1,0	0,0	0,0	1,0	1,0	1,0	0,0	0,0
yes	msc	derivate (window =9; polynomial = 2, degree = 1)	1,0	1,0	1,0	0,0	0,0	1,0	1,0	1,0	0,0	0,0
no	none	none	1,0	1,0	1,0	0,0	0,0	1,0	1,0	1,0	0,0	0,0
no	snv	derivate (window =9; polynomial = 2, degree = 2)	1,0	1,0	1,0	0,0	0,0	1,0	1,0	1,0	0,0	0,0
no	msc	derivate (window =9; polynomial = 2, degree = 2)	1,0	1,0	1,0	0,0	0,0	1,0	1,0	1,0	0,0	0,0
no	msc	derivate (window =15; polynomial = 2, degree = 0)	1,0	1,0	1,0	0,0	0,0	1,0	1,0	1,0	0,0	0,0
yes	snv	derivate (window =9; polynomial = 2, degree = 2)	1,0	1,0	1,0	0,0	0,0	1,0	1,0	1,0	0,0	0,0
yes	msc	derivate (window =9; polynomial = 2, degree = 2)	1,0	1,0	1,0	0,0	0,0	1,0	1,0	1,0	0,0	0,0

Pre-pretreatment			Train					Test				
**airPLS**	**Pretrat2**	**Pretrat3**	**ACC[1]**	**Sens.[2]**	**Spec[3]**	**FPR[4]**	**FNR[5]**	**ACC[1]**	**Sens.[2]**	**Spec[3]**	**FPR[4]**	**FNR[5]**
no	derivate (window =15; polynomial = 2, degree = 2)	none	1,0	1,0	1,0	0,0	0,0	1,0	1,0	1,0	0,0	0,0
yes	derivate (window =15; polynomial = 2, degree = 2)	none	1,0	1,0	1,0	0,0	0,0	1,0	1,0	1,0	0,0	0,0
yes	snv	derivate (window =15; polynomial = 2, degree = 2)	1,0	1,0	1,0	0,0	0,0	1,0	1,0	1,0	0,0	0,0
yes	msc	derivate (window =15; polynomial = 2, degree = 2)	1,0	1,0	1,0	0,0	0,0	1,0	1,0	1,0	0,0	0,0
no	snv	derivate (window =15; polynomial = 2, degree = 1)	1,0	1,0	1,0	0,0	0,0	1,0	1,0	1,0	0,0	0,0
no	snv	derivate (window =21; polynomial = 2, degree = 0)	1,0	1,0	1,0	0,0	0,0	1,0	1,0	1,0	0,0	0,0
no	msc	derivate (window =15; polynomial = 2, degree = 1)	1,0	1,0	1,0	0,0	0,0	1,0	1,0	1,0	0,0	0,0
no	derivate (window =9; polynomial = 2, degree = 1)	none	1,0	1,0	1,0	0,0	0,0	1,0	1,0	1,0	0,0	0,0
no	derivate (window =7; polynomial = 2, degree = 0)	none	1,0	1,0	1,0	0,0	0,0	1,0	1,0	1,0	0,0	0,0
no	msc	derivate (window =9; polynomial = 2, degree = 1)	1,0	1,0	1,0	0,0	0,0	1,0	1,0	1,0	0,0	0,0
no	msc	derivate (window =21; polynomial = 2, degree = 0)	1,0	1,0	1,0	0,0	0,0	1,0	1,0	1,0	0,0	0,0
yes	derivate (window =21; polynomial = 2, degree = 1)	none	1,0	1,0	1,0	0,0	0,0	1,0	1,0	1,0	0,0	0,0
no	snv	derivate (window =9; polynomial = 2, degree = 1)	1,0	1,0	1,0	0,0	0,0	1,0	0,9	1,0	0,0	0,1
no	snv	derivate (window =21; polynomial = 2, degree = 2)	1,0	1,0	1,0	0,0	0,0	1,0	0,9	1,0	0,0	0,1
no	msc	derivate (window =21; polynomial = 2, degree = 2)	1,0	1,0	1,0	0,0	0,0	1,0	1,0	1,0	0,0	0,0
no	derivate (window =21; polynomial = 2, degree = 0)	none	1,0	1,0	1,0	0,0	0,0	1,0	1,0	1,0	0,0	0,0
no	msc	derivate (window =21; polynomial = 2, degree = 1)	1,0	1,0	1,0	0,0	0,0	1,0	0,9	1,0	0,0	0,1

Pre-pretreatment			Train					Test				
**airPLS**	**Pretrat2**	**Pretrat3**	**ACC[1]**	**Sens.[2]**	**Spec[3]**	**FPR[4]**	**FNR[5]**	**ACC[1]**	**Sens.[2]**	**Spec[3]**	**FPR[4]**	**FNR[5]**
yes	snv	derivate (window =21; polynomial = 2, degree = 2)	1,0	1,0	1,0	0,0	0,0	0,9	0,8	1,0	0,0	0,2
yes	msc	derivate (window =21; polynomial = 2, degree = 2)	1,0	1,0	1,0	0,0	0,0	0,9	0,8	1,0	0,0	0,2
no	derivate (window =15; polynomial = 2, degree = 0)	none	1,0	1,0	1,0	0,0	0,0	0,9	0,8	1,0	0,0	0,2
no	derivate (window =21; polynomial = 2, degree = 1)	none	1,0	1,0	1,0	0,0	0,0	0,9	0,8	1,0	0,0	0,2
no	snv	derivate (window =21; polynomial = 2, degree = 1)	1,0	1,0	1,0	0,0	0,0	0,9	0,7	1,0	0,0	0,3
no	derivate (window =15; polynomial = 2, degree = 1)	none	1,0	1,0	1,0	0,0	0,0	0,9	0,7	1,0	0,0	0,3
no	derivate (window =21; polynomial = 2, degree = 2)	none	1,0	1,0	1,0	0,0	0,0	0,9	0,6	1,0	0,0	0,4
yes	derivate (window =21; polynomial = 2, degree = 2)	none	1,0	1,0	1,0	0,0	0,0	0,9	0,6	1,0	0,0	0,4

[1]Accuracy; [2]Sensitivity; [3]Specificity; [4]False Positive Rate ; [5]False Negative Rate.

Table S3. Performance parameters of the multiclass PLS-DA model using different pre-treatments for class 2 (250 mg.kg^{-1}).

Pre-pretreatment			Train					Test				
**airPLS**	**Pretrat2**	**Pretrat3**	**ACC[1]**	**Sens.[2]**	**Spec[3]**	**FPR[4]**	**FNR[5]**	**ACC[1]**	**Sens.[2]**	**Spec[3]**	**FPR[4]**	**FNR[5]**
no	svn	none	1,0	1,0	1,0	0,0	0,0	1,0	1,0	1,0	0,0	0,0
no	snv	derivate (window =7; polynomial = 2, degree = 0)	1,0	1,0	1,0	0,0	0,0	1,0	0,9	1,0	0,0	0,1
yes	snv	none	1,0	1,0	1,0	0,0	0,0	1,0	0,9	1,0	0,0	0,1
yes	norm	none	1,0	1,0	1,0	0,0	0,0	1,0	0,9	1,0	0,0	0,1
yes	derivate (window =15; polynomial = 2, degree = 1)	none	1,0	1,0	1,0	0,0	0,0	1,0	0,9	1,0	0,0	0,1
yes	derivate (window =15; polynomial = 2, degree = 0)	none	1,0	1,0	1,0	0,0	0,0	1,0	1,0	1,0	0,0	0,0
yes	derivate (window =21; polynomial = 2, degree = 0)	none	1,0	1,0	1,0	0,0	0,0	1,0	0,9	1,0	0,0	0,1
yes	snv	derivate (window =15; polynomial = 2, degree = 1)	1,0	1,0	1,0	0,0	0,0	1,0	0,9	1,0	0,0	0,1
yes	snv	derivate (window =7; polynomial = 2, degree = 0)	1,0	1,0	1,0	0,0	0,0	1,0	0,9	1,0	0,0	0,1
yes	snv	derivate (window =15; polynomial = 2, degree = 0)	1,0	1,0	1,0	0,0	0,0	1,0	0,9	1,0	0,0	0,1
yes	snv	derivate (window =21; polynomial = 2, degree = 0)	1,0	1,0	1,0	0,0	0,0	1,0	0,9	1,0	0,0	0,1
yes	msc	derivate (window =7; polynomial = 2, degree = 0)	1,0	1,0	1,0	0,0	0,0	0,9	0,8	1,0	0,0	0,2
yes	msc	derivate (window =15; polynomial = 2, degree = 0)	1,0	1,0	1,0	0,0	0,0	0,9	0,8	1,0	0,0	0,2
yes	msc	derivate (window =21; polynomial = 2, degree = 0)	1,0	1,0	1,0	0,0	0,0	0,9	0,8	1,0	0,0	0,2
no	snv	derivate (window =15; polynomial = 2, degree = 0)	1,0	1,0	1,0	0,0	0,0	0,9	0,8	1,0	0,0	0,2
yes	snv	derivate (window =21; polynomial = 2, degree = 1)	1,0	1,0	1,0	0,0	0,0	0,9	0,8	1,0	0,0	0,2
yes	msc	derivate (window =15; polynomial = 2, degree = 1)	1,0	1,0	1,0	0,0	0,0	0,9	0,8	1,0	0,0	0,2
yes	msc	derivate (window =21; polynomial = 2, degree = 1)	1,0	1,0	1,0	0,0	0,0	0,9	0,8	1,0	0,0	0,2

Pre-pretreatment			Train					Test				
airPLS	Pretrat2	Pretrat3	ACC[1]	Sens.[2]	Spec[3]	FPR[4]	FNR[5]	ACC[1]	Sens.[2]	Spec[3]	FPR[4]	FNR[5]
no	msc	none	1,0	1,0	1,0	0,0	0,0	0,9	0,8	1,0	0,0	0,2
no	norm	none	1,0	1,0	1,0	0,0	0,0	0,9	0,9	1,0	0,0	0,1
no	derivate (window =9; polynomial = 2, degree = 2)	none	1,0	1,0	1,0	0,0	0,0	0,9	0,8	1,0	0,0	0,2
no	snv	derivate (window =15; polynomial = 2, degree = 2)	1,0	1,0	1,0	0,0	0,0	0,9	0,8	1,0	0,0	0,2
no	msc	derivate (window =15; polynomial = 2, degree = 2)	1,0	1,0	1,0	0,0	0,0	0,9	0,8	1,0	0,0	0,2
no	msc	derivate (window =7; polynomial = 2, degree = 0)	1,0	1,0	1,0	0,0	0,0	0,9	0,7	1,0	0,0	0,3
yes	none	none	1,0	1,0	1,0	0,0	0,0	0,9	0,7	1,0	0,0	0,3
yes	msc	none	1,0	1,0	1,0	0,0	0,0	0,9	0,6	1,0	0,0	0,4
yes	derivate (window =9; polynomial = 2, degree = 1)	none	1,0	1,0	1,0	0,0	0,0	0,9	0,6	1,0	0,0	0,4
yes	derivate (window =9; polynomial = 2, degree = 2)	none	1,0	1,0	1,0	0,0	0,0	0,9	0,6	1,0	0,0	0,4
yes	derivate (window =7; polynomial = 2, degree = 0)	none	1,0	1,0	1,0	0,0	0,0	0,9	0,6	1,0	0,0	0,4
yes	snv	derivate (window =9; polynomial = 2, degree = 1)	1,0	1,0	1,0	0,0	0,0	0,9	0,6	1,0	0,0	0,4
yes	msc	derivate (window =9; polynomial = 2, degree = 1)	1,0	1,0	1,0	0,0	0,0	0,9	0,6	1,0	0,0	0,4
no	none	none	1,0	1,0	1,0	0,0	0,0	0,9	0,7	1,0	0,0	0,3
no	snv	derivate (window =9; polynomial = 2, degree = 2)	1,0	1,0	1,0	0,0	0,0	0,9	0,7	1,0	0,0	0,3
no	msc	derivate (window =9; polynomial = 2, degree = 2)	1,0	1,0	1,0	0,0	0,0	0,9	0,7	1,0	0,0	0,3
no	msc	derivate (window =15; polynomial = 2, degree = 0)	1,0	1,0	1,0	0,0	0,0	0,8	0,5	1,0	0,0	0,5
yes	snv	derivate (window =9; polynomial = 2, degree = 2)	1,0	1,0	1,0	0,0	0,0	0,8	0,5	1,0	0,0	0,5
yes	msc	derivate (window =9; polynomial = 2, degree = 2)	1,0	1,0	1,0	0,0	0,0	0,8	0,5	1,0	0,0	0,5

Pre-pretreatment			Train					Test				
airPLS	Pretrat2	Pretrat3	ACC[1]	Sens.[2]	Spec[3]	FPR[4]	FNR[5]	ACC[1]	Sens.[2]	Spec[3]	FPR[4]	FNR[5]
no	derivate (window =15; polynomial = 2, degree = 2)	none	1,0	1,0	1,0	0,0	0,0	0,9	0,6	1,0	0,0	0,4
yes	derivate (window =15; polynomial = 2, degree = 2)	none	1,0	1,0	1,0	0,0	0,0	0,9	0,8	0,9	0,1	0,2
yes	snv	derivate (window =15; polynomial = 2, degree = 2)	1,0	1,0	1,0	0,0	0,0	0,9	0,6	1,0	0,0	0,4
yes	msc	derivate (window =15; polynomial = 2, degree = 2)	1,0	1,0	1,0	0,0	0,0	0,8	0,5	1,0	0,0	0,5
no	snv	derivate (window =15; polynomial = 2, degree = 1)	1,0	1,0	1,0	0,0	0,0	0,9	0,7	1,0	0,0	0,3
no	snv	derivate (window =21; polynomial = 2, degree = 0)	1,0	1,0	1,0	0,0	0,0	0,9	0,6	1,0	0,0	0,4
no	msc	derivate (window =15; polynomial = 2, degree = 1)	1,0	1,0	1,0	0,0	0,0	0,9	0,7	1,0	0,0	0,3
no	derivate (window =9; polynomial = 2, degree = 1)	none	1,0	1,0	1,0	0,0	0,0	0,9	0,8	1,0	0,0	0,2
no	derivate (window =7; polynomial = 2, degree = 0)	none	1,0	1,0	1,0	0,0	0,0	0,9	0,7	0,9	0,1	0,3
no	msc	derivate (window =9; polynomial = 2, degree = 1)	1,0	1,0	1,0	0,0	0,0	0,9	0,7	0,9	0,1	0,3
no	msc	derivate (window =21; polynomial = 2, degree = 0)	1,0	1,0	1,0	0,0	0,0	0,9	0,7	1,0	0,0	0,3
yes	derivate (window =21; polynomial = 2, degree = 1)	none	1,0	1,0	1,0	0,0	0,0	0,9	0,8	1,0	0,0	0,2
no	snv	derivate (window =9; polynomial = 2, degree = 1)	1,0	1,0	1,0	0,0	0,0	0,9	0,9	0,9	0,1	0,1
no	snv	derivate (window =21; polynomial = 2, degree = 2)	1,0	1,0	1,0	0,0	0,0	0,9	0,9	0,9	0,1	0,1
no	msc	derivate (window =21; polynomial = 2, degree = 2)	1,0	1,0	1,0	0,0	0,0	0,9	0,8	1,0	0,1	0,2
no	derivate (window =21; polynomial = 2, degree = 0)	none	1,0	1,0	1,0	0,0	0,0	0,9	0,8	1,0	0,0	0,2
no	msc	derivate (window =21; polynomial = 2, degree = 1)	1,0	1,0	1,0	0,0	0,0	0,9	0,8	1,0	0,1	0,2

Pre-pretreatment			Train					Test				
**airPLS**	**Pretrat2**	**Pretrat3**	**ACC[1]**	**Sens.[2]**	**Spec[3]**	**FPR[4]**	**FNR[5]**	**ACC[1]**	**Sens.[2]**	**Spec[3]**	**FPR[4]**	**FNR[5]**
yes	snv	derivate (window =21; polynomial = 2, degree = 2)	1,0	1,0	1,0	0,0	0,0	0,9	0,9	1,0	0,0	0,1
yes	msc	derivate (window =21; polynomial = 2, degree = 2)	1,0	1,0	1,0	0,0	0,0	0,9	0,9	1,0	0,0	0,1
no	derivate (window =15; polynomial = 2, degree = 0)	none	1,0	1,0	1,0	0,0	0,0	0,8	0,4	0,9	0,1	0,6
no	derivate (window =21; polynomial = 2, degree = 1)	none	1,0	1,0	1,0	0,0	0,0	0,8	0,4	0,9	0,1	0,6
no	snv	derivate (window =21; polynomial = 2, degree = 1)	1,0	1,0	1,0	0,0	0,0	0,9	0,9	0,9	0,1	0,1
no	derivate (window =15; polynomial = 2, degree = 1)	none	1,0	1,0	1,0	0,0	0,0	0,9	0,9	0,9	0,1	0,1
no	derivate (window =21; polynomial = 2, degree = 2)	none	1,0	1,0	1,0	0,0	0,0	0,8	0,8	0,8	0,2	0,2
yes	derivate (window =21; polynomial = 2, degree = 2)	none	1,0	1,0	1,0	0,0	0,0	0,8	0,8	0,8	0,2	0,2

[1]Accuracy; [2]Sensitivity; [3]Specificity; [4]False Positive Rate ; [5]False Negative Rate.

Table S4. Performance parameters of the multiclass PLS-DA model using different pre-treatments for class 3 (500 mg.kg^{-1}).

Pre-pretreatment			Train					Test				
**airPLS**	**Pretrat2**	**Pretrat3**	**ACC[1]**	**Sens.[2]**	**Spec[3]**	**FPR[4]**	**FNR[5]**	**ACC[1]**	**Sens.[2]**	**Spec[3]**	**FPR[4]**	**FNR[5]**
no	svn	none	1,0	1,0	1,0	0,0	0,0	1,0	1,0	1,0	0,0	0,0
no	snv	derivate (window =7; polynomial = 2, degree = 0)	1,0	1,0	1,0	0,0	0,0	1,0	1,0	1,0	0,0	0,0
yes	snv	none	1,0	1,0	1,0	0,0	0,0	1,0	1,0	1,0	0,1	0,0
yes	norm	none	1,0	1,0	1,0	0,0	0,0	1,0	1,0	1,0	0,0	0,0
yes	derivate (window =15; polynomial = 2, degree = 1)	none	1,0	1,0	1,0	0,0	0,0	1,0	1,0	1,0	0,0	0,0
yes	derivate (window =15; polynomial = 2, degree = 0)	none	1,0	1,0	1,0	0,0	0,0	1,0	0,9	1,0	0,0	0,1
yes	derivate (window =21; polynomial = 2, degree = 0)	none	1,0	1,0	1,0	0,0	0,0	1,0	1,0	1,0	0,0	0,0
yes	snv	derivate (window =15; polynomial = 2, degree = 1)	1,0	1,0	1,0	0,0	0,0	1,0	1,0	1,0	0,1	0,0
yes	snv	derivate (window =7; polynomial = 2, degree = 0)	1,0	1,0	1,0	0,0	0,0	1,0	1,0	1,0	0,0	0,0
yes	snv	derivate (window =15; polynomial = 2, degree = 0)	1,0	1,0	1,0	0,0	0,0	1,0	1,0	1,0	0,0	0,0
yes	snv	derivate (window =21; polynomial = 2, degree = 0)	1,0	1,0	1,0	0,0	0,0	1,0	1,0	1,0	0,0	0,0
yes	msc	derivate (window =7; polynomial = 2, degree = 0)	1,0	1,0	1,0	0,0	0,0	0,9	1,0	0,9	0,1	0,0
yes	msc	derivate (window =15; polynomial = 2, degree = 0)	1,0	1,0	1,0	0,0	0,0	0,9	1,0	0,9	0,1	0,0
yes	msc	derivate (window =21; polynomial = 2, degree = 0)	1,0	1,0	1,0	0,0	0,0	0,9	1,0	0,9	0,1	0,0
no	snv	derivate (window =15; polynomial = 2, degree = 0)	1,0	1,0	1,0	0,0	0,0	0,9	1,0	0,9	0,1	0,0
yes	snv	derivate (window =21; polynomial = 2, degree = 1)	1,0	1,0	1,0	0,0	0,0	0,9	1,0	0,9	0,1	0,0
yes	msc	derivate (window =15; polynomial = 2, degree = 1)	1,0	1,0	1,0	0,0	0,0	0,9	1,0	0,9	0,1	0,0
yes	msc	derivate (window =21; polynomial = 2, degree = 1)	1,0	1,0	1,0	0,0	0,0	0,9	1,0	0,9	0,1	0,0

Pre-pretreatment			Train					Test				
**airPLS**	**Pretrat2**	**Pretrat3**	**ACC**[1]	**Sens.**[2]	**Spec**[3]	**FPR**[4]	**FNR**[5]	**ACC**[1]	**Sens.**[2]	**Spec**[3]	**FPR**[4]	**FNR**[5]
no	msc	none	1,0	1,0	1,0	0,0	0,0	0,9	1,0	0,9	0,1	0,0
no	norm	none	1,0	1,0	1,0	0,0	0,0	0,9	0,9	1,0	0,0	0,1
no	derivate (window =9; polynomial = 2, degree = 2)	none	1,0	1,0	1,0	0,0	0,0	0,9	1,0	0,9	0,1	0,0
no	snv	derivate (window =15; polynomial = 2, degree = 2)	1,0	1,0	1,0	0,0	0,0	0,9	1,0	0,9	0,1	0,0
no	msc	derivate (window =15; polynomial = 2, degree = 2)	1,0	1,0	1,0	0,0	0,0	0,9	1,0	0,9	0,1	0,0
no	msc	derivate (window =7; polynomial = 2, degree = 0)	1,0	1,0	1,0	0,0	0,0	0,9	1,0	0,9	0,1	0,0
yes	none	none	1,0	1,0	1,0	0,0	0,0	0,9	1,0	0,9	0,2	0,0
yes	msc	none	1,0	1,0	1,0	0,0	0,0	0,9	1,0	0,9	0,1	0,0
yes	derivate (window =9; polynomial = 2, degree = 1)	none	1,0	1,0	1,0	0,0	0,0	0,9	1,0	0,9	0,1	0,0
yes	derivate (window =9; polynomial = 2, degree = 2)	none	1,0	1,0	1,0	0,0	0,0	0,9	1,0	0,9	0,1	0,0
yes	derivate (window =7; polynomial = 2, degree = 0)	none	1,0	1,0	1,0	0,0	0,0	0,9	1,0	0,9	0,1	0,0
yes	snv	derivate (window =9; polynomial = 2, degree = 1)	1,0	1,0	1,0	0,0	0,0	0,9	1,0	0,9	0,1	0,0
yes	msc	derivate (window =9; polynomial = 2, degree = 1)	1,0	1,0	1,0	0,0	0,0	0,9	0,9	0,9	0,1	0,1
no	none	none	1,0	1,0	1,0	0,0	0,0	0,9	0,9	0,9	0,1	0,1
no	snv	derivate (window =9; polynomial = 2, degree = 2)	1,0	1,0	1,0	0,0	0,0	0,9	0,9	0,9	0,1	0,1
no	msc	derivate (window =9; polynomial = 2, degree = 2)	1,0	1,0	1,0	0,0	0,0	0,9	0,9	0,9	0,1	0,1
no	msc	derivate (window =15; polynomial = 2, degree = 0)	1,0	1,0	1,0	0,0	0,0	0,8	1,0	0,8	0,2	0,0
yes	snv	derivate (window =9; polynomial = 2, degree = 2)	1,0	1,0	1,0	0,0	0,0	0,8	1,0	0,8	0,2	0,0
yes	msc	derivate (window =9; polynomial = 2, degree = 2)	1,0	1,0	1,0	0,0	0,0	0,8	1,0	0,7	0,3	0,0

Pre-pretreatment			Train					Test				
airPLS	Pretrat2	Pretrat3	ACC[1]	Sens.[2]	Spec[3]	FPR[4]	FNR[5]	ACC[1]	Sens.[2]	Spec[3]	FPR[4]	FNR[5]
no	derivate (window =15; polynomial = 2, degree = 2)	none	1,0	1,0	1,0	0,0	0,0	0,9	0,9	0,9	0,1	0,1
yes	derivate (window =15; polynomial = 2, degree = 2)	none	1,0	1,0	1,0	0,0	0,0	0,9	0,6	1,0	0,0	0,4
yes	snv	derivate (window =15; polynomial = 2, degree = 2)	1,0	1,0	1,0	0,0	0,0	0,9	1,0	0,9	0,1	0,0
yes	msc	derivate (window =15; polynomial = 2, degree = 2)	1,0	1,0	1,0	0,0	0,0	0,9	1,0	0,8	0,2	0,0
no	snv	derivate (window =15; polynomial = 2, degree = 1)	1,0	1,0	1,0	0,0	0,0	0,9	1,0	0,9	0,1	0,0
no	snv	derivate (window =21; polynomial = 2, degree = 0)	1,0	1,0	1,0	0,0	0,0	0,9	1,0	0,9	0,1	0,0
no	msc	derivate (window =15; polynomial = 2, degree = 1)	1,0	1,0	1,0	0,0	0,0	0,9	1,0	0,9	0,1	0,0
no	derivate (window =9; polynomial = 2, degree = 1)	none	1,0	1,0	1,0	0,0	0,0	0,9	1,0	0,9	0,1	0,0
no	derivate (window =7; polynomial = 2, degree = 0)	none	1,0	1,0	1,0	0,0	0,0	0,9	1,0	0,9	0,1	0,0
no	msc	derivate (window =9; polynomial = 2, degree = 1)	1,0	1,0	1,0	0,0	0,0	0,9	1,0	0,9	0,1	0,0
no	msc	derivate (window =21; polynomial = 2, degree = 0)	1,0	1,0	1,0	0,0	0,0	0,9	1,0	0,8	0,2	0,0
yes	derivate (window =21; polynomial = 2, degree = 1)	none	1,0	1,0	1,0	0,0	0,0	0,9	0,8	1,0	0,0	0,2
no	snv	derivate (window =9; polynomial = 2, degree = 1)	1,0	1,0	1,0	0,0	0,0	0,9	1,0	0,9	0,1	0,0
no	snv	derivate (window =21; polynomial = 2, degree = 2)	1,0	1,0	1,0	0,0	0,0	1,0	1,0	1,0	0,0	0,0
no	msc	derivate (window =21; polynomial = 2, degree = 2)	1,0	1,0	1,0	0,0	0,0	0,9	0,8	1,0	0,0	0,2
no	derivate (window =21; polynomial = 2, degree = 0)	none	1,0	1,0	1,0	0,0	0,0	0,9	0,7	1,0	0,0	0,3
no	msc	derivate (window =21; polynomial = 2, degree = 1)	1,0	1,0	1,0	0,0	0,0	0,9	1,0	0,8	0,2	0,0

Pre-pretreatment			Train					Test				
**airPLS**	**Pretrat2**	**Pretrat3**	**ACC[1]**	**Sens.[2]**	**Spec[3]**	**FPR[4]**	**FNR[5]**	**ACC[1]**	**Sens.[2]**	**Spec[3]**	**FPR[4]**	**FNR[5]**
yes	snv	derivate (window =21; polynomial = 2, degree = 2)	1,0	1,0	1,0	0,0	0,0	1,0	1,0	1,0	0,0	0,0
yes	msc	derivate (window =21; polynomial = 2, degree = 2)	1,0	1,0	1,0	0,0	0,0	1,0	1,0	1,0	0,0	0,0
no	derivate (window =15; polynomial = 2, degree = 0)	none	1,0	1,0	1,0	0,0	0,0	0,8	0,8	0,9	0,1	0,3
no	derivate (window =21; polynomial = 2, degree = 1)	none	1,0	1,0	1,0	0,0	0,0	0,8	0,8	0,9	0,1	0,3
no	snv	derivate (window =21; polynomial = 2, degree = 1)	1,0	1,0	1,0	0,0	0,0	1,0	1,0	1,0	0,0	0,0
no	derivate (window =15; polynomial = 2, degree = 1)	none	1,0	1,0	1,0	0,0	0,0	1,0	1,0	1,0	0,0	0,0
no	derivate (window =21; polynomial = 2, degree = 2)	none	1,0	1,0	1,0	0,0	0,0	1,0	1,0	1,0	0,0	0,0
yes	derivate (window =21; polynomial = 2, degree = 2)	none	1,0	1,0	1,0	0,0	0,0	1,0	1,0	1,0	0,0	0,0

[1]Accuracy; [2]Sensitivity; [3]Specificity; [4]False Positive Rate ; [5]False Negative Rate.

Table S5. Performance parameters of the multiclass PLS-DA model using different pre-treatments for class 4 (1000 mg.kg^{-1}).

Pre-pretreatment			Train					Test				
**airPLS**	**Pretrat2**	**Pretrat3**	**ACC[1]**	**Sens.[2]**	**Spec[3]**	**FPR[4]**	**FNR[5]**	**ACC[1]**	**Sens.[2]**	**Spec[3]**	**FPR[4]**	**FNR[5]**
no	svn	none	1,0	1,0	1,0	0,0	0,0	1,0	1,0	1,0	0,0	0,0
no	snv	derivate (window =7; polynomial = 2, degree = 0)	1,0	1,0	1,0	0,0	0,0	1,0	1,0	1,0	0,0	0,0
yes	snv	none	1,0	1,0	1,0	0,0	0,0	1,0	1,0	1,0	0,0	0,0
yes	norm	none	1,0	1,0	1,0	0,0	0,0	1,0	1,0	1,0	0,0	0,0
yes	derivate (window =15; polynomial = 2, degree = 1)	none	1,0	1,0	1,0	0,0	0,0	1,0	1,0	1,0	0,0	0,0
yes	derivate (window =15; polynomial = 2, degree = 0)	none	1,0	1,0	1,0	0,0	0,0	1,0	1,0	1,0	0,0	0,0
yes	derivate (window =21; polynomial = 2, degree = 0)	none	1,0	1,0	1,0	0,0	0,0	1,0	1,0	1,0	0,0	0,0
yes	snv	derivate (window =15; polynomial = 2, degree = 1)	1,0	1,0	1,0	0,0	0,0	1,0	1,0	1,0	0,0	0,0
yes	snv	derivate (window =7; polynomial = 2, degree = 0)	1,0	1,0	1,0	0,0	0,0	1,0	1,0	1,0	0,0	0,0
yes	snv	derivate (window =15; polynomial = 2, degree = 0)	1,0	1,0	1,0	0,0	0,0	1,0	1,0	1,0	0,0	0,0
yes	snv	derivate (window =21; polynomial = 2, degree = 0)	1,0	1,0	1,0	0,0	0,0	1,0	1,0	1,0	0,0	0,0
yes	msc	derivate (window =7; polynomial = 2, degree = 0)	1,0	1,0	1,0	0,0	0,0	1,0	1,0	1,0	0,0	0,0
yes	msc	derivate (window =15; polynomial = 2, degree = 0)	1,0	1,0	1,0	0,0	0,0	1,0	1,0	1,0	0,0	0,0
yes	msc	derivate (window =21; polynomial = 2, degree = 0)	1,0	1,0	1,0	0,0	0,0	1,0	1,0	1,0	0,0	0,0
no	snv	derivate (window =15; polynomial = 2, degree = 0)	1,0	1,0	1,0	0,0	0,0	1,0	1,0	1,0	0,0	0,0
yes	snv	derivate (window =21; polynomial = 2, degree = 1)	1,0	1,0	1,0	0,0	0,0	1,0	1,0	1,0	0,0	0,0
yes	msc	derivate (window =15; polynomial = 2, degree = 1)	1,0	1,0	1,0	0,0	0,0	1,0	1,0	1,0	0,0	0,0
yes	msc	derivate (window =21; polynomial = 2, degree = 1)	1,0	1,0	1,0	0,0	0,0	1,0	1,0	1,0	0,0	0,0

Pre-pretreatment			Train					Test				
airPLS	Pretrat2	Pretrat3	ACC[1]	Sens.[2]	Spec[3]	FPR[4]	FNR[5]	ACC[1]	Sens.[2]	Spec[3]	FPR[4]	FNR[5]
no	msc	none	1,0	1,0	1,0	0,0	0,0	1,0	1,0	1,0	0,0	0,0
no	norm	none	1,0	1,0	1,0	0,0	0,0	1,0	1,0	1,0	0,0	0,0
no	derivate (window =9; polynomial = 2, degree = 2)	none	1,0	1,0	1,0	0,0	0,0	1,0	1,0	1,0	0,0	0,0
no	snv	derivate (window =15; polynomial = 2, degree = 2)	1,0	1,0	1,0	0,0	0,0	1,0	1,0	1,0	0,0	0,0
no	msc	derivate (window =15; polynomial = 2, degree = 2)	1,0	1,0	1,0	0,0	0,0	1,0	1,0	1,0	0,0	0,0
no	msc	derivate (window =7; polynomial = 2, degree = 0)	1,0	1,0	1,0	0,0	0,0	1,0	1,0	1,0	0,0	0,0
yes	none	none	1,0	1,0	1,0	0,0	0,0	1,0	1,0	1,0	0,0	0,0
yes	msc	none	1,0	1,0	1,0	0,0	0,0	1,0	1,0	1,0	0,0	0,0
yes	derivate (window =9; polynomial = 2, degree = 1)	none	1,0	1,0	1,0	0,0	0,0	1,0	1,0	1,0	0,0	0,0
yes	derivate (window =9; polynomial = 2, degree = 2)	none	1,0	1,0	1,0	0,0	0,0	1,0	1,0	1,0	0,0	0,0
yes	derivate (window =7; polynomial = 2, degree = 0)	none	1,0	1,0	1,0	0,0	0,0	1,0	1,0	1,0	0,0	0,0
yes	snv	derivate (window =9; polynomial = 2, degree = 1)	1,0	1,0	1,0	0,0	0,0	1,0	1,0	1,0	0,0	0,0
yes	msc	derivate (window =9; polynomial = 2, degree = 1)	1,0	1,0	1,0	0,0	0,0	1,0	1,0	1,0	0,0	0,0
no	none	none	1,0	1,0	1,0	0,0	0,0	1,0	1,0	1,0	0,0	0,0
no	snv	derivate (window =9; polynomial = 2, degree = 2)	1,0	1,0	1,0	0,0	0,0	1,0	1,0	1,0	0,0	0,0
no	msc	derivate (window =9; polynomial = 2, degree = 2)	1,0	1,0	1,0	0,0	0,0	1,0	1,0	1,0	0,0	0,0
no	msc	derivate (window =15; polynomial = 2, degree = 0)	1,0	1,0	1,0	0,0	0,0	1,0	1,0	1,0	0,0	0,0
yes	snv	derivate (window =9; polynomial = 2, degree = 2)	1,0	1,0	1,0	0,0	0,0	1,0	1,0	1,0	0,0	0,0
yes	msc	derivate (window =9; polynomial = 2, degree = 2)	1,0	1,0	1,0	0,0	0,0	1,0	1,0	1,0	0,0	0,0

Pre-pretreatment			Train					Test				
airPLS	Pretrat2	Pretrat3	ACC[1]	Sens.[2]	Spec[3]	FPR[4]	FNR[5]	ACC[1]	Sens.[2]	Spec[3]	FPR[4]	FNR[5]
no	derivate (window =15; polynomial = 2, degree = 2)	none	1,0	1,0	1,0	0,0	0,0	1,0	1,0	1,0	0,0	0,0
yes	derivate (window =15; polynomial = 2, degree = 2)	none	1,0	1,0	1,0	0,0	0,0	1,0	1,0	1,0	0,0	0,0
yes	snv	derivate (window =15; polynomial = 2, degree = 2)	1,0	1,0	1,0	0,0	0,0	1,0	0,9	1,0	0,0	0,1
yes	msc	derivate (window =15; polynomial = 2, degree = 2)	1,0	1,0	1,0	0,0	0,0	1,0	0,9	1,0	0,0	0,1
no	snv	derivate (window =15; polynomial = 2, degree = 1)	1,0	1,0	1,0	0,0	0,0	1,0	0,9	1,0	0,0	0,1
no	snv	derivate (window =21; polynomial = 2, degree = 0)	1,0	1,0	1,0	0,0	0,0	1,0	0,9	1,0	0,0	0,1
no	msc	derivate (window =15; polynomial = 2, degree = 1)	1,0	1,0	1,0	0,0	0,0	1,0	0,8	1,0	0,0	0,2
no	derivate (window =9; polynomial = 2, degree = 1)	none	1,0	1,0	1,0	0,0	0,0	1,0	0,8	1,0	0,0	0,2
no	derivate (window =7; polynomial = 2, degree = 0)	none	1,0	1,0	1,0	0,0	0,0	1,0	0,8	1,0	0,0	0,2
no	msc	derivate (window =9; polynomial = 2, degree = 1)	1,0	1,0	1,0	0,0	0,0	0,9	0,8	1,0	0,0	0,2
no	msc	derivate (window =21; polynomial = 2, degree = 0)	1,0	1,0	1,0	0,0	0,0	0,9	0,7	1,0	0,0	0,3
yes	derivate (window =21; polynomial = 2, degree = 1)	none	1,0	1,0	1,0	0,0	0,0	1,0	1,0	1,0	0,0	0,0
no	snv	derivate (window =9; polynomial = 2, degree = 1)	1,0	1,0	1,0	0,0	0,0	1,0	0,5	1,0	0,0	0,5
no	snv	derivate (window =21; polynomial = 2, degree = 2)	1,0	1,0	1,0	0,0	0,0	1,0	1,0	1,0	0,0	0,0
no	msc	derivate (window =21; polynomial = 2, degree = 2)	1,0	1,0	1,0	0,0	0,0	1,0	1,0	1,0	0,0	0,0
no	derivate (window =21; polynomial = 2, degree = 0)	none	1,0	1,0	1,0	0,0	0,0	1,0	1,0	1,0	0,0	0,0
no	msc	derivate (window =21; polynomial = 2, degree = 1)	1,0	1,0	1,0	0,0	0,0	1,0	0,7	1,0	0,0	0,3

Pre-pretreatment			Train					Test				
airPLS	Pretrat2	Pretrat3	ACC[1]	Sens.[2]	Spec[3]	FPR[4]	FNR[5]	ACC[1]	Sens.[2]	Spec[3]	FPR[4]	FNR[5]
yes	snv	derivate (window =21; polynomial = 2, degree = 2)	1,0	1,0	1,0	0,0	0,0	1,0	1,0	1,0	0,0	0,0
yes	msc	derivate (window =21; polynomial = 2, degree = 2)	1,0	1,0	1,0	0,0	0,0	1,0	1,0	1,0	0,0	0,0
no	derivate (window =15; polynomial = 2, degree = 0)	none	1,0	1,0	1,0	0,0	0,0	1,0	1,0	1,0	0,0	0,0
no	derivate (window =21; polynomial = 2, degree = 1)	none	1,0	1,0	1,0	0,0	0,0	1,0	1,0	1,0	0,0	0,0
no	snv	derivate (window =21; polynomial = 2, degree = 1)	1,0	1,0	1,0	0,0	0,0	1,0	1,0	1,0	0,0	0,0
no	derivate (window =15; polynomial = 2, degree = 1)	none	1,0	1,0	1,0	0,0	0,0	1,0	1,0	1,0	0,0	0,0
no	derivate (window =21; polynomial = 2, degree = 2)	none	1,0	1,0	1,0	0,0	0,0	1,0	1,0	1,0	0,0	0,0
yes	derivate (window =21; polynomial = 2, degree = 2)	none	1,0	1,0	1,0	0,0	0,0	1,0	1,0	1,0	0,0	0,0

[1]Accuracy; [2]Sensitivity; [3]Specificity; [4]False Positive Rate ; [5]False Negative Rate.
